# Humanization of care in Primary Health Care: social representations of people with pulmonary tuberculosis*

**DOI:** 10.1590/1980-220X-REEUSP-2025-0008en

**Published:** 2026-01-26

**Authors:** Mayara Del Aguilal Pacheco, Iací Proença Palmeira, Márcia de Assunção Ferreira, Aloma Sena Soares, Erlon Gabriel Rego de Andrade, Alexandre Aguiar Pereira, Ivaneide Leal Ataíde Rodrigues, Widson Davi Vaz de Matos

**Affiliations:** 1 Universidade Federal do Rio de Janeiro, Escola de Enfermagem Anna Nery, Programa de Pós-Graduação em Enfermagem, Rio de Janeiro, RJ, Brazil.; 2 Universidade do Estado do Pará, Escola de Enfermagem Magalhães Barata, Programa de Pós-Graduação em Enfermagem, Belém, PA, Brazil.

**Keywords:** Humanization of Assistance, Primary Health Care, Tuberculosis, Pulmonary, Social Representation, Psychology, Social

## Abstract

**Objective:**

To analyze the social representations of people with pulmonary tuberculosis regarding humanization of care in Primary Healthcare.

**Method:**

This descriptive, qualitative study is based on the Social Representation Theory’s procedural approach. It was conducted in four Family Health Units in Belém, Pará, Brazil, with 30 individuals undergoing treatment for pulmonary tuberculosis. Between March and June 2022, individual semi-structured interviews were conducted, the *corpus* of which was submitted to the *Analyse Lexicale par Contexte d’un Ensemble de Segments de Texte* (version 2012) software.

**Results:**

The *corpus* was divided into 470 Elementary Context Units, using 363 (77.23%). Five lexical classes were generated, with classes 2 and 3 standing out for highlighting the social representations of the topic. This context supported humanization of care by a sense of trust and good treatment, despite the limitations of the physical structure and material resources of the health units.

**Conclusion:**

Human relationships are important in care activities, especially given the challenges of multidisciplinary work in Primary Healthcare, encouraging the development and strengthening of bonds between teams and patients.

## INTRODUCTION

Popularly known as tuberculosis or the white plague, tuberculosis (TB) is an ancient disease characterized by its infectious, preventable, and curable nature. Its etiology is associated with infection by *Mycobacterium tuberculosis* bacilli, which, when expelled by a person carrying the bacterium when sneezing, speaking, or coughing, can infect ten to 15 individuals in a community. It manifests in two clinical forms: pulmonary TB, which affects the lungs, and extrapulmonary TB, which affects other organs and systems^(1,2)^.

Worldwide, in 2023, it was estimated that 10.8 million people fell ill and 1.25 million died from TB^(1)^. In Brazil, 80,012 new cases were reported and, in 2022, 5,845 deaths, resulting in incidence and mortality rates of 37.3 cases per 100,000 inhabitants and 2.8 deaths per 100,000 inhabitants. In the state of Pará, considering the same years, 4,293 new cases and 341 deaths were recorded, ranking first in absolute number of new cases and deaths from the disease in northern Brazil, with rates of 48.8 cases per 100,000 inhabitants and 3.9 deaths per 100,000 inhabitants. In turn, in the municipality of Belém, there were 1,360 new cases and 135 deaths, with rates of 90.7 cases per 100,000 inhabitants and 9.0 deaths per 100,000 inhabitants^(3)^.

It is known that the links between healthcare professionals and users are fundamental to qualifying care and promoting effective control of the disease as a public health concern^(4)^. The Brazilian National Humanization Policy (In Portuguese, *Política Nacional de Humanização* – PNH) supported this by establishing by the Ministry of Health in 2003, aiming to improve care in the Brazilian Health System (In Portuguese, *Sistema Único de Saúde* – SUS) to provide more welcoming and participatory actions, which is why it presents, as one of its principles, users’ and workers’ leading role. To achieve a comprehensive and humanized approach, it proposes a care model that values qualified listening, co-responsibility, and increased dialogue between professionals and users in the therapeutic process, respecting human dignity and equitable access to healthcare services^(5)^.

In the care of people with TB, the PNH plays a crucial role in fostering practices that overcome fragmented care and ensure continuous monitoring, which is essential for patients to adhere to long-term antimicrobial medication use. Successful PNH implementation requires participatory management structures that ensure, among other elements, user participation in clinical decision-making, their leading role in demanding humane attitudes and behaviors from professionals, continuing training of professionals, and a decisive dialogue among participants^(5)^. Approaches that value dialogue, connections, and comprehensiveness provide personalized and effective care, reducing stigmas and other sociocultural barriers that often limit therapeutic success among people with TB^(6,7)^.

Humanization is also expressed as a relational phenomenon, with implications for developing good relationships in the workplace and strengthening bonds between professionals and users, favoring provision of care centered on the specificities of those affected, who demand a holistic view so that their biopsychosocial needs are met^(8)^. In the daily lives of people with TB, treated in Primary Health Care (PHC) units, quality care actions become possible when there is a good interpersonal relationship between them and professionals during the therapeutic process, culminating in the construction or strengthening of users’ feeling of trust^(9,10)^.

However, fear and other negative feelings are still evident among certain professionals when they learn that users have TB, reactivating the social memory of stigma and prejudice that distances them from those affected, compromising bonds^(11)^. This occurs because these professionals express mistaken representations about how TB is transmitted, indicating a possible lack of qualified information that is capable of influencing their actions in the therapeutic monitoring of those affected^(12)^.

It is also understood that lack of information can generate unpleasant feelings among people with TB, fueling negative representations that arise from stigmatization and social exclusion, considering that the disease is related to beliefs and taboos wrapped in feelings such as fear and sadness in the face of diagnosis and treatment^(9)^. Thus, the absence or lack of care that is compassionate, empathetic, and responsive to the needs of those affected contributes to the insufficient performance of interpersonal relationships or even to non-adherence to treatment^(13,14)^.

The limitations of humanization in PHC must be treated as a solvable problem, the resolution of which is necessary for the proper functioning of the SUS^(8)^. In this context, it is essential to combine technical and scientific knowledge with professionals’ humane actions for the appropriate management and control of TB. This weighting is made considering that PHC constitutes the first level of care recommended for people with TB, a setting in which it is possible to mobilize knowledge, feelings, and behaviors that bring those affected closer to or further away from PHC services, impacting both the individual and collective levels^(15)^.

Research on the topic points to the Social Representation Theory (SRT), developed by psychologist Serge Moscovici, which focuses on social representations (SRs) as forms of particular knowledge that organize various knowledge in the cognitive field and originate from communications and group relationships. SRs are formed by two memory-based cognitive processes: anchoring, which integrates a new or unusual social object into previous mental constructs; and objectification, which materializes this object with images or symbols from daily sociocultural life. They present three dimensions: informational, which involves the construction and structuring of knowledge arising from interactions and communications; attitude, which refers to individuals’ judgment or positioning regarding the object; and field of representation or image dimension, which relates to other components associated with the object, following the logic of a social model, in which the elements of SRs are ordered and hierarchized^(16)^.

In this context, two logics of thought organize and express SRs: consensual universe, defined by the set of common-sense knowledge; and reified knowledge, a set of technical knowledge originating in science. By studying SRs, it is possible to comprehend how and why people know, think, and act in certain ways when faced with social phenomena that circulate in their daily lives^(16)^, which is especially relevant when it comes to humanization of care in TB control.

Practices that correspond to humanized care are characterized by elements such as sensitive listening and quality communication, and promote meaningful bonds between professionals and users, impacting their way of acting and adherence or not to health recommendations^(5,8)^. These practices are constructed from a psychosociological perspective, marked by the interaction between the person, the professional and the environment that surrounds them, approaching SRT by showing that knowledge about an object is closely related to subjects’ actions^(15,17)^.

PHC is a favorable setting for building, disseminating and strengthening SRs, as interactions shape daily actions^(15,17)^. Therefore, comprehending the SRs of people with TB regarding humanization, at the first level of care, can reveal how social thinking influences behaviors according to the meanings attributed during the therapeutic process.

Considering the relevance of the topic, the guiding question was formulated: how do people with pulmonary TB represent humanization of care in PHC? To answer this question, this study aimed to analyze the social representations of people with pulmonary tuberculosis regarding humanization of care in Primary Health Care.

## METHOD

### Study Design

This descriptive research, with a qualitative approach, is based on the SRT’s procedural approach. This theory was chosen because it allows us to investigate how individuals construct explanations about a given social object and interpret it through language and the relationships between the object and socio-historical and cultural conditions, enabling us to comprehend the actions adopted in daily life^(16,17)^. This study followed the COnsolidated criteria for REporting Qualitative research (COREQ) guide recommendations^(18)^.

### Locations

The study took place in four Family Health Units (FHUs) located in the Sacramenta Administrative District (In Portuguese, *Distrito Administrativo da Sacramenta* – DASAC), one of the eight administrative districts of the municipality of Belém, Pará state, Brazil. A previous survey conducted at the Belém Municipal Health Department (In Portuguese, *Secretaria Municipal de Saúde de Belém* – SESMA) found that, in 2021, the DASAC registered a large number of pulmonary TB cases: approximately 126 (40.13%) of the 314 registered in the municipality. In the same year, the selected units presented the highest number of cases under monitoring in this district, justifying their selection.

### Population and Selection Criteria

People over 18 years old, who received at least two months of treatment for pulmonary TB, and registered with one of the selected FHUs were included. The two-month period was determined based on participants’ ambiance and familiarity with healthcare services’ routines and TB treatment. These criteria are fundamental for assimilating the SRs on humanization of care, as they configure sociocultural applicability through the immersion of the object in individuals’ daily life^(16)^. People with communication difficulties detected by the main researcher, given that these difficulties could limit participation in the interviews, were excluded.

The population consisted of 70 individuals being monitored at the four FHUs. Applying the criteria, 34 eligible individuals were invited, three of whom declined and one was excluded due to communication difficulties caused by an acute mental disorder. There were no dropouts. Thus, with non-probability sampling, the sample consisted of 30 participants, representing 42.86% of the total. This number met theoretical saturation, as the preliminary analysis of statements revealed that the data produced were sufficient to comprehend the phenomenon and achieve the objective, as recommended in the qualitative approach^(19)^.

### Data Collection

Data were collected between March and June 2022 through individual interviews conducted by the lead researcher, a master’s student in nursing trained in orientation meetings with the second and third authors and in regular meetings of a research group focused primarily on developing qualitative studies, whether or not coordinated with the SRT. During preliminary visits, she introduced herself to the managers of the FHUs and the multidisciplinary teams working there, aiming to facilitate the identification of people with pulmonary TB and to arrange a room for the interviews, respecting participants’ privacy and comfort without interfering with services’ routines.

As participants arrived at the units, the researcher approached them individually after the appointments and invited them to participate, directing those who agreed to a private room. In this room, occupied only by the researcher and the participant, the objectives, procedures, risks, and benefits were presented and explained in accessible language to facilitate understanding and obtain formal acceptance.

Each interview was guided by a semi-structured script, created by the authors, with nine closed-ended questions to characterize participant sociodemographic and epidemiological profile according to the variables age, sex, marital status, religion, education, insertion in the labor market, monthly family income, health unit in which they were followed up, and type of admission (new case, relapse, re-entry after abandonment or transfer), whose answers were manually recorded in the printed version.

Furthermore, it consisted of open-ended questions to explore the phenomenon, covering aspects such as knowledge about humanization of care for people with TB and other groups, relationships with professionals working in the units, and guidance experiences based on the information they shared. The responses to these questions were audio-recorded in MP3 format, using an electronic device, and lasted approximately 30 to 40 minutes.

The script was not subjected to a pilot test, but was assessed by four professors with doctoral degrees who researched various topics in the fields of nursing and public health, who endorsed its content, making it possible to achieve the objective with in-depth interviews, without needing to repeat them and without requiring other production techniques.

### Data Analysis and Treatment

Sociodemographic and epidemiological profile data were stored in a Microsoft Office Excel^®^ spreadsheet (version 2021) and treated using descriptive statistics to calculate relative numbers from absolute numbers. The responses to the open-ended questions were transcribed in full to construct the *corpus* and submitted, in a single file, to lexical analysis using the *Analyse Lexicale par Contexte d’un Ensemble de Segments de Texte* (Alceste^®^, version 2012) software, which processes textual data with sophisticated statistical methods for lexicographic analysis. This allows for a careful interpretation of the analyzed texts, tracking the vocabularies (lexicons) used by participants, as well as their occurrence, co-occurrence, and statistical association (*Phi*)^(20)^.

In Alceste^®^, the text of each interview is called an Initial Context Unit (ICU), and the set of ICUs forms the *corpus*. This software organizes words into dendrograms of lexical classes, according to their stems, and the meanings of these classes are captured in Elementary Context Units (ECUs), which correspond to the excerpts selected by the software from the analyzed ICUs^(20)^.

Specifically, the Descending Hierarchical Classification was considered, organized in descending order, respecting both the index of statistical association of stems (*Phi*) with the classes and their corresponding ECUs. The data were interpreted and discussed based on pertinent evidence from scientific literature on the subject and SRT precepts, presented in the introduction in light of Serge Moscovici’s^(16)^ and Denise Jodelet’s^(17)^ ideas, the main theorist of the procedural aspect.

### Ethical Aspects

In compliance with the ethical precepts of Resolution 466/2012 of the Brazilian National Health Council, the research was authorized by SESMA and approved by the *Universidade do Estado do Pará* Research Ethics Committee, under Opinion 5,214,278, issued in January 2022. All participants signed the Informed Consent Form prior to the interviews, declaring their formal and voluntary acceptance. The confidentiality of their identities was preserved by using alphanumeric codes formed by the ICU acronym, followed by a hyphen and a cardinal number, which indicate the order of the interviews.

## RESULTS

Of the 30 participants, 22 (73.33%) were between 18 and 49 years old, of which 19 (63.33%) were male. Furthermore, 14 (46.67%) were married or living together in a consensual union; 15 (50%) were evangelical; 11 (36.67%) reported incomplete elementary education, and 11 (36.67%), complete high school education; 11 (36.67%) were unemployed; 19 (63.33%) had a monthly family income between one and three minimum wages; and 26 (86.67%) were new cases of TB.

Using Alceste^®^, the *corpus* was segmented into 470 ECUs, composed of 2,345 distinct words, resulting in the use of 363 ECUs (77.23%), considering all the content of the ICUs. Five lexical classes were generated, with classes 2 and 3 standing out as they highlight the SR on humanization of care. Therefore, they will be presented here, along with some emblematic excerpts that demonstrate their characteristic meanings, enabling the study objective to be met.

Class 2 consisted of 60 ECUs (16.53%) and 66 analyzable words. Class 3, in turn, had 76 ECUs (20.94%) and 84 analyzable words. Chart 1 presents the stems, full words, and corresponding *Phi* values assigned by Alceste^®^. To organize the results, the authors assigned the titles “Humanized assistance based on interpersonal relationships” and “Obstacles to the practice of humanization”, respectively to these classes, considering their contents.

**Chart 1:** Descending Hierarchical Classification of classes 2 and 3, with roots, full words, and their *Phi* values – Belém, PA, Brazil, 2022.

Class 2			Class 3		
Stems	Full words	*Phi*	Stems	Full words	*Phi*
Listen	Listen	0.33	Assist	Assisted	0.32
Care	Care	0.31	Deliver	Deliver, delivered, delivering, delivery	0.26
Profession	Professional, professionals	0.29	Complain	Complain, complained, complaining, complaint	0.26
Treat	Treat, treated	0.28	Space	Space	0.25
Happen	Happen, happened, happens	0.26	Queue	Queue, queues	0.24
Patient	Patient, patients	0.26	Need	Need, needed, needing	0.24
Humanization of ca	Humanization_of_care	0.26	Receive	Receive, received	0.22
Human care	Humanized_care	0.25	Structure	Structure	0.22
Ques	Question, questioning, questions	0.23	End	End, ended, ending	0.22
Give	Give	0.23	Assist	Assist, assisted	0.22
Listen	Listen	0.23	Patient	Patient, patients	0.22
Comment	Comment, comments	0.23	Drink	Drink	0.20
Arrog	Arrogance	0.23	Place	Place, Places	0.20
Hear	Hear, heard, hearing	0.20	Receiv	Received	0.19

### Class 2 – Humanized Assistance Based on Interpersonal Relationships

The most significant stems of this class and the ECUs that gave them meaning pointed out that humanized care is expressed in the interpersonal relationships that occur in PHC, and the comprehension of the phenomenon of humanization was anchored in daily experiences, mainly through good treatment:

*I’ve never heard of humanizing care! Maybe it’s about whether the care is good or not. I think good care is when nurses treat us well, without ignorance, because there are some places that treat us with arrogance.* (ICU-1)

*I think that humanized care is when a doctor treats their patients well and patients agree with their words.* (ICU-2)

Good service, translated as good treatment, was understood through elements that qualify attitudes and behaviors in interpersonal relationships, based on professionals’ attentive attitude, conversations, empathy, and patience:

*The professionals here treat everyone equally and are very attentive. During my visits here, the care I’ve always received has been excellent; I have no complaints.* (ICU-6)

*Being well assisted is when professionals talk to patients without arrogance, and even if the health unit is not of quality, they do what they can to make patients feel good.* (ICU-23)

In this regard, qualified listening and interest in a person with TB were cited as fundamental elements for them to feel welcomed. However, when this did not occur in certain situations in participants’ experience, the relationships between professionals and patients were interpreted as discriminatory actions, marked by a notable lack of interest:

*In other places, professionals don’t pay attention, they just write things down and don’t want to listen to how a patient is feeling. The nurse at this health center calls me, texts me, and checks in on me, and that’s good.* (ICU-10)

*I’ve never been treated indifferently at this clinic, but at the first clinic where I sought care, yes. I went with a social worker to register, and she didn’t listen to me or ask where the nearest place to get care was. She sent me to this clinic with the wrong referral form, handed me a tissue, and told me to put on another mask when I already had two.* (ICU-20)

For this reason, the welcoming of patients and the understanding of their reality by professionals helped characterize humanized care. By interpreting humanized care through the prism of professional care, they viewed professionals as respectful, kind, and not confusing their personal and professional lives:

*For this type of care to occur, a good professional is necessary, because there are some who mix personal problems with work, and are unable to provide humanized care.* (ICU-13)

[...] *there needs to be professionals who understand and comprehend the situation of people who come seeking healthcare, because not everyone provides good care and welcome.* (ICU-18)

### Class 3 – Obstacles to the Practice of Humanization

In this class, the stems that indicated the most significant words gained meaning in the ECUs that revealed obstacles to the practice of humanization, such as limitations in the physical structure of health units and the lack of material resources, which compromised professionals’ work and, consequently, care provided to users:

*The space is too small to accommodate the large number of patients. There aren’t enough chairs for everyone, and it’s very hot.* (ICU-11)

*The building’s physical structure and material resources need to be improved so that professionals can perform their work better and, therefore, patients are benefited.* (ICU-22)

Participants comprehended that improving services depended on investments by public managers, including improvements in physical space and the compatibility of human resources in relation to the number of users, approaching reified knowledge to develop their SRs:

*What still needs improvement is related to the municipality’s responsibility. For instance, adding more doctors to care for patients and improving the space, which is still small, for a population that grows every day.* (ICU-I7)

*I think the government could invest more to improve the health center’s physical structure and security, expand the space and provide more professionals to provide services.* (ICU-23)

Considering the routine of actions to control TB in these units, they stated that adherence to treatment depended on the way they were treated by professionals, so that, if they felt disrespected or were treated with prejudice, they would abandon treatment and seek it elsewhere, strongly evidencing the consensual universe to judge the viability of this adherence:

*If I noticed any prejudiced behavior from professionals here, I certainly wouldn’t come back and would give up treatment or continue somewhere else.* (ICU-22)

*If I had received poor treatment, I would have definitely stopped treatment here, looked for another place, or taken home remedies.* (ICU-27)

## DISCUSSION

The statements revealed that, in their attempt to explain the phenomenon, participants represented humanization based on interpersonal relationships, grounded in the idea of good care, and also based on its opposite, “dehumanization”, which was anchored in daily experiences, including the structural problems of the health facilities where they received care. Greater concern from public management is needed to implement the PNH. In this context, a close relationship was identified between reified knowledge and the consensual universe.

Evidenced by participants’ profile, group belonging is an important element of studies based on SRT, as it allows us to understand the context from which they express their representations, which Jodelet calls the “lifeworld”^(17)^. It was found, for instance, that the predominant age group was close to the TB Epidemiological Bulletin results, published in 2024, which indicated the age groups of 20 to 34 and 35 to 49 years as the majority in males, and 20 to 34 years, in females^(3)^. The prevalence among men follows the global trend of TB illness^(1,3)^, also supported by other studies^(13,14)^.

Unemployment data points to a worrying scenario, as treatment is long, costly, and requires adequate nutrition. For those without income, meeting the physiological needs arising from the disease and the therapeutic process, including nutritional needs, can be even more challenging, resulting in possible abandonment or insufficient response to treatment due to malnutrition. In view of this, the Ministry of Health of Brazil has promoted social protection initiatives for individuals and families affected by TB, considering the close relationship between poverty and disease. Thus, it carries out intersectoral actions involving health, education, and social assistance, aiming for the well-being of those affected through comprehensive care^(6)^.

Health and education actions are developed through care practices carried out by multidisciplinary teams. These practices should coordinate care for people with TB with care for those with whom they share the illness (family members, caregivers, and companions), focusing on aspects such as treatment, prevention of complications, rehabilitation, and social reintegration of those affected, but also active search, assessment, and monitoring of clinical conditions, disease prevention, and, if necessary, diagnosis, treatment, and rehabilitation among contacts^(2)^.

Depending on the biopsychosocial needs of those affected and their contacts, this requires the joint participation of these sectors, as it is known that educational level and conditions influence the natural history of socially determined diseases such as TB, favoring or reducing individual and collective willingness and capabilities to comprehend and incorporate self-care and prevention practices, making them elements that confirm the importance of health education. Furthermore, it is through education that human resources are qualified to work in TB control, which is why systematic actions must be proposed and implemented to encourage continuing education and permanent education, aiming to address the limitations arising from health work or mitigate their effects^(2)^.

Weaving this explanation in light of SRT implies remembering that SRs are inserted in a pre-existing context full of traditions, values, and beliefs that are anchored to the object that is represented, which is incorporated into the daily explanations of individuals and their groups. Such composition or exchange of knowledge/experiences is essential to build thoughts based on the consensual universe and to share them with the group, providing opportunities for social belonging^(16)^.

Access to social support and income transfer programs, such as *Aposentadoria para Pessoas de Baixa Renda* (Retirement for Low-Income People), *Benefício de Prestação Continuada* (Continuous Benefit Payment), *Programa Bolsa Família* (Family Allowance), *Minha Casa/Minha Vida* (My House/My Life), and *Tarifa Social de Energia Elétrica* (Social Electricity Tariff), in force in Brazil, can mitigate the socioeconomic repercussions of TB on the family budget and strengthen quality of life^(21,22)^. Although they are not specific to people with the disease, it is understood that knowledge about these programs and the possibility of implementing them are fundamental to qualifying assistance, especially for low-income people^(23)^. Aiming at the collective fight against TB, this reinforces the coordination that must exist between the SUS and the Unified Social Assistance System (In Portuguese, *Sistema Único de Assistência Social* – SUAS), as reiterated by the Ministry of Health and the Ministry of Citizenship, when publishing Operational Instruction 1 of September 26, 2019^(24)^.

Regarding SRs, anchored in a set of common-sense categories and the understanding of the therapeutic process as good or poor, participants constructed their knowledge about humanization based on oppositions, with this understanding organized through the reality experienced in the care received. This occurs because the paradigms stored in social memory establish a positive or negative relationship with their inherent content, which is accessed through cognitive activity^(16)^.

The word “humanization” is conceptually difficult to comprehend, but when associated with experiences in healthcare services (informational dimension), participants demonstrated an understanding that treating others well and opting for a fruitful relationship (attitude dimension) generates positive behaviors in the social context (field of representation or image dimension). Thus, SR’s three dimensions are identified^(16)^, structuring the phenomenon of humanization associated with healthcare.

In this context, participants embodied humanization based on the good care professionals should provide, characterized by elements such as qualified listening, welcoming, empathy, and respect, widely desired in services’ daily routine at all levels of care (primary, secondary/specialized, and tertiary/hospital). Other studies showed similar results, when users anchored humanization to the healthcare team’s good practices, desiring to be treated with affection, politeness, and good humor, mentioned as actions that, in their view, identified good care^(25,26)^.

Cognition and affection are intertwined in the construction of knowledge and learning processes, as human development is mediated by culture as well as by emotions and social interactions^(27)^. Therefore, it is assumed that the connections between cognitive, affective, and social aspects influence the construction of knowledge that produces meanings about a given object^(16,17)^, which is why, in this study, the SR moved dialectically between the dehumanization and humanization of care for people with TB, as several PNH elements were cited, sometimes by their presence, sometimes by their absence.

For this reason, dialogue without arrogance is a necessary condition for establishing horizontal relationships, facilitating the comprehending of information shared between people. This is addressed in the PNH and, therefore, should generate the set of actions known as welcoming, essential to caring relationships, as it encompasses, among other aspects, the sharing of information and the human connection to guide pertinent and timely decisions^(8)^. Similarly, other studies point to welcoming relationships as a humanized expression that facilitates the creation of bonds and a feeling of trust, helping to solve daily problems^(8,28)^.

It was under this understanding that participants objectified healthcare professionals as respectful, kind, and not confusing their personal life with the life they pursued in their profession, from the perspective of being people who welcomed and were able to address patients’ concerns. The shift from the abstract, from the idea-concept, to the figurative model is part of objectification, and this model’s function is to present a common point between different knowledges, translate the real representation of the object, and associate the elements in a dynamic that is unique to it^(16)^.

The long period of TB treatment and follow-up in PHC allows for the creation of horizontal bonds, but it is essential that professionals and patients engage in strengthening their interpersonal relationships, which should be based on five pillars: cordiality; ethics; empathy; assertiveness; and self-knowledge. The cordiality pillar refers to being kind and helpful, while the ethics pillar refers to a sense of justice, principle, and discipline, both necessary to improve care and make it effective in addressing health problems, in this case, TB^(29)^.

The empathy pillar is inherent in the act of putting oneself in another person’s shoes, being willing to listen to them, a context in which the results of this research showed that qualified listening is an essential component of care practices^(29)^. Listening to others can occur at various times, such as during reception, which fosters timely dialogue between professionals and patients, tailored to their needs, enabling the assessment of vulnerabilities, severity, and risks, fostering commitment and bonding among these stakeholders. This allows for increased effectiveness of healthcare practices, contributing to an expanded clinical practice by fostering shared decision-making committed to SUS patients’ autonomy^(5)^.

From this perspective, the representation of humanized care through two human cognitive actions, “comprehending” and “understanding”, mentioned by ICU-18 in class 2, drew attention. Comprehending is an ongoing process; therefore, when addressing this concept, it is assumed that there is no such thing as something defined and immutable, as what exists is a comprehensibility in constant transformation. Understanding, in turn, consists of listening, perceiving, and assimilating the purpose of something^(30)^.

Thus, comprehending implies a highly engaging action, permeated by a commitment to knowledge about the deepest aspects of the subject, while understanding configures a more superficial process and derives from the word “understand”. Interpreting humanized care through both words highlights that this type of care is imbued with characteristics specific to interaction. Even if the institutional structure is sophisticated and the environment welcoming, nothing surpasses the presence of a professional, as only a human being is capable of comprehending another^(30)^. Therefore, when users make a cognitive effort to represent humanized care in light of their experiences, comprehending becomes a prominent element that translates the human relationship, so necessary for care^(31)^.

To comprehend others, it is necessary to establish contact to help them overcome adversities that can interfere with self-care, treatment, and healing. Changing perspectives on lifestyle habits that can lead to illness or slow healing often requires the necessary (re)classification of daily actions, including relationships and social interactions. This requires professionals to establish bonds of trust with patients so that, through the construction of a horizontal dialogue, care becomes effective^(5,30)^.

The assertiveness pillar strengthens interpersonal relationships when it addresses interactions between professionals and patients, based on clear, direct, and respectful communication. In turn, the self-knowledge pillar allows professionals to comprehend their personality to avoid or mitigate conflicts^(29)^, as indicated in the results of this study, where participants recognized the importance of separating personal and professional life aspects.

When it comes to humanization, it is worth highlighting that, without adequate initiatives for permanent education, there is no satisfactory quality of care practices^(5)^. Therefore, referring to the need for personal problems not to interfere with good service, participants noted that professionals should have not only technical-scientific capacity, but also relational skills and abilities to combat dehumanizing actions, which impacted the ability to comprehend users.

Another important aspect concerns the environment elements, as they strongly influence care and, consequently, treatment and healing. Analyzing the PNH, it is clear that the environment is a humanizing instrument, as it should help promote comfort, safety, and well-being, in addition to being an element that integrates the multidisciplinary team, patients, and their families^(25)^.

Considering the specificities of TB, the Ministry of Health recommends that, in PHC, there be correct architectural planning of health units to prevent the spread of *Mycobacterium tuberculosis* and other airborne pathogens, valuing items such as adequate lighting and ventilation, since, in the case of TB, the bacilli expelled by people with the disease can disperse quickly^(2)^. Therefore, it is essential that there are spaces whose characteristics guarantee comfort for these people and health safety for other groups that frequent them, enabling the breaking of the chain of transmission.

The municipality of Belém is characterized by a hot and humid climate, with periods of little or a lot of rain^(32)^. Therefore, small spaces in PHC units, especially in the daily activities to control TB, can significantly interfere with human health, given that closed environments encourage the dispersion of bacilli^(2)^ and the circulation of particles and small organisms, such as dust and mites^(33)^.

According to the PNH, the concept of ambiance necessarily incorporates welcoming, comfortable spaces that guarantee the privacy of human groups, but that also favor, whenever necessary, changes in the work process, promoting meetings based on dialogue^(5)^. Therefore, these spaces must offer professionals and users an adequate and interactive environment.

In contrast, the statements revealed a small space with few chairs, insufficient to meet the demand of those seeking care. This reality hindered the multidisciplinary work process and emotional relationships, reducing patients’ well-being in the care setting.

This consideration is made because, in addition to the representations that circulate about TB, there are the challenges faced by subjects. These challenges are presented in a cumulative manner and result in negative and/or positive aspects in the face of experiences surrounding the disease, allowing the construction of new perspectives and meanings that will be beneficial or harmful, strengthening or limiting, necessary or unnecessary for daily life and for coping with TB, depending on the nature of their contents^(34)^.

Thus, participants identified public management as responsible for delivering service improvements through effective investments in PHC. In this regard, they acknowledged that unit teams implemented health actions, but it was up to managers to provide the necessary resources. Therefore, the lack of basic resources to improve services was characterized as an inhumane condition, as it impacted the health of both professionals and users.

This means that dehumanization can be evidenced not only through attitudes and behaviors, but also through the lack of structure and resources, which hinder therapeutic adherence among people with TB^(28)^. This comprehending helps explain participants’ decision to seek other services or opt for other forms of treatment if the care received at the units was disrespectful or showed signs of prejudice.

From this perspective, the interlocution between reified knowledge and the consensual universe enables the dynamism of SRs, a context in which changes in conception and the union of knowledge that comes from both logics of thought are fundamental to (re)construct these SRs. It also makes it possible to comprehend how scientific knowledge is appropriated by people in social discourse and becomes common content of popular knowledge, providing elements to characterize the ways in which social groups think and act when faced with an object or context of subjective importance^(35)^.

This result highlights that the SRs on humanized care, in the context of TB, is based on the idea of good care as a benchmark for maintaining users’ follow-up. This is an important element, as it demonstrates that adequate investment in professionals’ technical-scientific and relational training is essential for effective treatment^(2)^.

For the PNH, humanizing care means valuing the different subjects involved in the health production process (users, workers and managers), including values such as autonomy and leading role, co-responsibility, establishment of solidary bonds, and collective participation in the management process^(31)^. A well-functioning health system, with participatory management, financing, workforce, and technological resources, encourages the progress of PHC^(36)^.

Therefore, it is necessary to improve the PNH and expand the practices of its professionals, developing alternatives that value differences, singularities, and humanized practices. These practices must be supported by attitudes based on mutual respect, but also by actions focused on collective commitment. It is worth highlighting that, in the context of health, structural and sociocultural changes, aimed at the quality and humanization of care, can become important elements in the process of expanding the symbolic field of SRs on humanization^(37)^.

The limitations of this study stem from its implementation in a specific municipality within a Brazilian state, which restricts the scope of its results to settings with similar or approximate characteristics. Further SRs studies on the topic are needed to encompass all three levels of care. Therefore, it is crucial to comprehend this topic in other social settings, helping to develop and disseminate reflections based on the equal appreciation of reified knowledge and the consensual universe – thinking logics that enable the identification of SRs.

Given its results, this study contributes to nursing and public health, as it can support fruitful dialogue between students, professionals, managers, and public authorities to improve healthcare services and propose new strategies that strengthen humanization in the daily care of people with TB.

This consideration is made considering that, in a practical context, these social actors can be encouraged to think critically about the factors that hinder the practice of humanization in PHC services. Consistent reflective practice can guide them in proposing measures that impact the various elements involved in humanization, including the structural characteristics of health units, multidisciplinary work processes, and the human relationships they forge with users, as highlighted in the statements. To be effective and strengthen humanization, such measures must consider the administrative, material, operational, political, and sociocultural challenges inherent to these services, aiming to transform their realities to some degree.

## CONCLUSION

It was evident that humanization of care was anchored in interpersonal relationships established between healthcare professionals and patients, sustained by the sense of trust and good treatment characteristic of qualified care. However, in participants’ reality, it is possible to infer that this was not achieved due to the limitations of the physical structure of the health units where they received care and the lack of material resources, recognized as problems inherent to public management that needed to be overcome to strengthen TB control actions in PHC.

Thus, interpersonal relationships were the cornerstone of humanization, facilitating mutual comprehending of the disease, the therapeutic process, and the adversities that could occur in this context, fostering treatment adherence. Thus, knowing the elements that constituted or influenced the social responses of people with TB on the topic demonstrated the importance of human relationships in care activities, especially given the challenges of multidisciplinary work in PHC, encouraging the development and strengthening of bonds between teams and patients.

Given the SRs revealed here, it is understood that new research can help comprehend other psychosocial aspects surrounding humanization, resulting in contributions to care, service management, teaching, and research in the health field. These spheres of power and sociopolitical action are inherent to various professionals, including nurses, whose ethical, social, and technical responsibilities guide the identification of human groups’ needs to implement interventions that transform, to some degree, the reality they experience, enabling the incorporation of individualized care practices.

## Data Availability

The data supporting this study are available upon request to the corresponding author.
