# Supplementary material for: Humanization of care in Primary Health Care: social representations of people with pulmonary tuberculosis
Source: Rev Esc Enferm USP. 2026 Jan 26;60:e20250008. doi: 10.1590/1980-220X-REEUSP-2025-0008en (PMC12839317; doi:10.1590/1980-220X-REEUSP-2025-0008en)
Supplement: Translation

# Translation: Humanização da assistência na Atenção Primária à Saúde: representações sociais de pessoas com tuberculose pulmonar*

**DOI:** 10.1590/1980-220X-REEUSP-2025-0008pt

**Authors:** Mayara Del Aguilal Pacheco, Iací Proença Palmeira, Márcia de Assunção Ferreira, Aloma Sena Soares, Erlon Gabriel Rego de Andrade, Alexandre Aguiar Pereira, Ivaneide Leal Ataíde Rodrigues, Widson Davi Vaz de Matos

**Affiliations:** 1 Universidade Federal do Rio de Janeiro, Escola de Enfermagem Anna Nery, Programa de Pós-Graduação em Enfermagem, Rio de Janeiro, RJ, Brasil.; 2 Universidade do Estado do Pará, Escola de Enfermagem Magalhães Barata, Programa de Pós-Graduação em Enfermagem, Belém, PA, Brasil.

**Keywords:** Humanização da Assistência, Atenção Primária à, Saúde, Tuberculose Pulmonar, Representação Social, Psicologia Social

## Abstract

**Objetivo:**

Analisar as representações sociais de pessoas com tuberculose pulmonar sobre a humanização da assistência na Atenção Primária à Saúde.

**Método:**

Pesquisa descritiva, com abordagem qualitativa, fundamentada na vertente processual da Teoria das Representações Sociais. Foi realizada em quatro Unidades de Saúde da Família, em Belém, Pará, Brasil, com 30 pessoas em tratamento para tuberculose pulmonar. Entre março e junho de 2022, realizaram-se entrevistas individuais semiestruturadas, cujo *corpus* foi submetido ao *software Analyse Lexicale par Contexte d’un Ensemble de Segments de Texte* (versão 2012).

**Resultados:**

O *corpus* foi dividido em 470 Unidades de Contexto Elementar, aproveitando-se 363 (77,23%). Foram geradas cinco classes lexicais, destacando-se as classes 2 e 3 por evidenciarem as representações sociais acerca do tema, contexto no qual a humanização da assistência foi sustentada pelo sentimento de confiança e pelo bom trato, apesar das limitações na estrutura física e nos recursos materiais das unidades de saúde.

**Conclusão:**

As relações humanas são importantes nas atividades assistenciais, sobretudo perante os desafios do trabalho multiprofissional na Atenção Primária à Saúde, incentivando a construção e o fortalecimento de vínculos entre equipes e usuários.

## INTRODUÇÃO

Doença milenar, popularmente conhecida como tísica ou peste branca, a tuberculose (TB) é caracterizada por ser infectocontagiosa, prevenível e curável. Sua etiologia é associada à infecção por bacilos da espécie *Mycobacterium tuberculosis* que, ao serem expelidos por pessoa bacilífera ao espirrar, falar ou tossir, podem contaminar de dez a 15 indivíduos em uma comunidade. Expressa duas formas clínicas, denominadas TB pulmonar, cujos órgãos afetados são os pulmões, e TB extrapulmonar, que afeta outros órgãos e sistemas^(1,2)^.

No mundo, em 2023, estimou-se que 10,8 milhões de pessoas adoeceram e 1,25 milhão morreram de TB^(1)^. No Brasil, foram notificados 80.012 casos novos e, em 2022, 5.845 óbitos, resultando nos coeficientes de incidência e de mortalidade iguais a 37,3 casos/100 mil habitantes e 2,8 óbitos/100 mil habitantes. No estado do Pará, considerando os mesmos anos, foram registrados 4.293 casos novos e 341 óbitos, ocupando o primeiro lugar em número absoluto de casos novos e óbitos da doença na região Norte do país, com coeficientes de 48,8 casos/100 mil habitantes e 3,9 óbitos/100 mil habitantes. Por sua vez, no município de Belém, ocorreram 1.360 casos novos e 135 óbitos, com coeficientes de 90,7 casos/100 mil habitantes e 9,0 óbitos/100 mil habitantes^(3)^.

Sabe-se que os vínculos entre profissionais de saúde e usuários são fundamentais para qualificar o cuidado e promover o controle efetivo da doença como problema de saúde pública^(4)^. Corroborando isso, a Política Nacional de Humanização (PNH), instituída pelo Ministério da Saúde em 2003, objetiva aprimorar os atendimentos no Sistema Único de Saúde (SUS) para proporcionar ações mais acolhedoras e participativas, motivo pelo qual apresenta, como um dos seus princípios, o protagonismo dos usuários e dos trabalhadores. Para alcançar uma abordagem integral e humanizada, propõe um modelo de atenção que valoriza a escuta qualificada, a corresponsabilidade e a ampliação do diálogo entre profissionais e usuários no processo terapêutico, respeitando a dignidade humana e o acesso equitativo aos serviços de saúde^(5)^.

Na assistência às pessoas com TB, a PNH desempenha papel crucial ao fomentar práticas que superem a fragmentação do cuidado e assegurem o acompanhamento contínuo, essencial para que os acometidos adiram ao uso de medicamentos antimicrobianos por longo período. A implementação bem sucedida da PNH requer estruturas de gestão participativa que assegurem, entre outros elementos, a participação dos usuários na tomada de decisão clínica, o seu protagonismo para exigir que profissionais tenham atitudes e comportamentos humanizados, a capacitação contínua dos profissionais, e o diálogo resolutivo entre os partícipes^(5)^. Abordagens que valorizam o diálogo, os vínculos e a integralidade propiciam ações de cuidado personalizadas e eficazes, reduzindo estigmas e outras barreiras socioculturais que, frequentemente, limitam o sucesso terapêutico entre pessoas com TB^(6,7)^.

A humanização também se expressa como fenômeno relacional, com implicações para desenvolver boas relações no ambiente de trabalho e fortalecer os vínculos entre profissionais e usuários, favorecendo a prestação de cuidados centrados nas especificidades dos acometidos, os quais demandam um olhar holístico para que suas necessidades biopsicossociais sejam atendidas^(8)^. No cotidiano das pessoas com TB, atendidas em unidades da Atenção Primária à Saúde (APS), ações de cuidado com qualidade se tornam possíveis quando há bom relacionamento interpessoal entre elas e os profissionais durante o processo terapêutico, culminando na construção ou no fortalecimento do sentimento de confiança por parte do usuário^(9,10)^.

Contudo, ainda se evidenciam o medo e outros afetos negativos entre certos profissionais ao saberem que o usuário está com TB, reativando a memória social do estigma e do preconceito que os afastam dos acometidos, comprometendo os vínculos^(11)^. Isso ocorre devido a esses profissionais manifestarem representações equivocadas sobre o modo de transmitir a TB, indicando possível carência de informações qualificadas e que sejam capazes de influenciar suas ações no acompanhamento terapêutico dos acometidos^(12)^.

Entende-se, também, que a carência de informações pode gerar sentimentos desagradáveis entre pessoas com TB, alimentando representações negativas que advêm da estigmatização e exclusão social, considerando que a doença se relaciona a crenças e tabus envoltos em afetos, como medo e tristeza perante o diagnóstico e tratamento^(9)^. Assim, a ausente ou carente prestação de cuidados que sejam compassivos, empáticos e responsivos às necessidades dos acometidos contribui para a performance insuficiente das relações interpessoais ou mesmo para a não adesão ao tratamento^(13,14)^.

As limitações da humanização na APS devem ser tratadas como problema solucionável, cuja resolução é necessária para o bom funcionamento do SUS^(8)^. Nesse contexto, é fundamental associar conhecimentos técnico-científicos às ações humanizadas dos profissionais para o adequado manejo e controle da TB. Faz-se tal ponderação considerando que a APS configura o primeiro nível de atenção recomendado para atender pessoas com TB, cenário no qual é possível mobilizar saberes, afetos e comportamentos que aproximem ou afastem os acometidos em relação aos serviços da APS, repercutindo nos âmbitos individual e coletivo^(15)^.

Investigações acerca do tema apontam para a Teoria das Representações Sociais (TRS), elaborada pelo psicólogo Serge Moscovici, com foco nas representações sociais (RS) como formas de conhecimento particular que organizam vários saberes no campo cognitivo e são originadas a partir das comunicações e dos relacionamentos em grupo. As RS são formadas por dois processos cognitivos baseados na memória: a ancoragem, que integra um novo ou inusitado objeto social às construções mentais prévias; e a objetivação, que materializa esse objeto com imagens ou símbolos do cotidiano sociocultural. Apresentam três dimensões: a informação, que envolve a construção e estruturação de saberes provenientes das interações e comunicações; a atitude, que se refere ao juízo ou posicionamento do indivíduo diante do objeto; e o campo representacional ou dimensão da imagem, que se relaciona com outros componentes associados ao objeto, seguindo a lógica de um modelo social, em que os elementos das RS são ordenados e hierarquizados^(16)^.

Nesse contexto, duas lógicas de pensamento organizam e expressam as RS: o universo consensual, definido pelo conjunto de saberes do senso comum; e o conhecimento reificado, conjunto de saberes técnicos originados na ciência. Ao estudar as RS, é possível compreender como e por que as pessoas sabem, pensam e agem de certa maneira diante de fenômenos sociais que circulam no seu cotidiano^(16)^, o que é especialmente relevante se tratando da humanização da assistência no controle da TB.

As práticas que correspondem à assistência humanizada são caracterizadas por elementos como escuta sensível e comunicação de qualidade, e promovem vínculos significativos entre profissionais e usuários, repercutindo no seu modo de agir e na adesão ou não às recomendações de saúde^(5,8)^. Essas práticas são construídas sob uma perspectiva psicossociológica, marcada pela interação entre a pessoa, o profissional e o ambiente que os circunda, aproximando-se da TRS ao mostrar que os saberes sobre um objeto estão intimamente relacionados às ações dos sujeitos^(15,17)^.

A APS é um cenário propício para construir, disseminar e fortalecer RS, visto que as interações moldam as ações cotidianas^(15,17)^. Portanto, conhecer as RS de pessoas com TB sobre a humanização, no primeiro nível de atenção, pode revelar como o pensamento social influencia comportamentos de acordo com os sentidos atribuídos durante o processo terapêutico.

Considerando a relevância do tema, formulou-se a questão norteadora: como pessoas com TB pulmonar representam a humanização da assistência na APS? Para respondê-la, este estudo teve como objetivo analisar as representações sociais de pessoas com tuberculose pulmonar sobre a humanização da assistência na Atenção Primária à Saúde.

## MÉTODO

### Tipo de Estudo

Pesquisa descritiva, com abordagem qualitativa, fundamentada na vertente processual da TRS. Optou-se por essa teoria considerando que permite investigar como os sujeitos constroem explicações sobre determinado objeto social e o interpretam por meio da linguagem e das relações entre o objeto e as condições sócio-históricas e culturais, possibilitando compreender as ações adotadas no cotidiano^(16,17)^. Foram seguidas as recomendações do guia *COnsolidated criteria for REporting Qualitative research* (COREQ)^(18)^.

### Locais

Ocorreu em quatro Unidades de Saúde da Família (USFs) localizadas no Distrito Administrativo da Sacramenta (DASAC), um dos oito distritos administrativos do município de Belém, estado do Pará, Brasil. Em levantamento prévio, realizado na Secretaria Municipal de Saúde de Belém (SESMA), verificou-se que, em 2021, o DASAC apresentou grande número de casos cadastrados de TB pulmonar: cerca de 126 (40,13%), do total de 314 registrados no município. No mesmo ano, as unidades selecionadas apresentaram os maiores números de casos em acompanhamento nesse distrito, justificando a escolha por elas.

### População e Critérios de Seleção

Para selecionar os participantes, os critérios de inclusão foram ser maior de 18 anos, com, no mínimo, dois meses de tratamento para TB pulmonar, e estar cadastrado em uma das USFs selecionadas. A delimitação do período de dois meses considerou a ambiência e convivência do participante com as rotinas dos serviços de saúde e o tratamento da TB, critérios fundamentais para apreender as RS sobre a humanização da assistência, pois configuram aplicabilidade sociocultural pela imersão do objeto no cotidiano do sujeito^(16)^. Os critérios de exclusão foram ser pessoas com dificuldades de comunicação detectadas pela pesquisadora principal, haja vista que essas dificuldades poderiam limitar a participação nas entrevistas.

A população se constituiu por 70 pessoas em acompanhamento nas quatro USFs. Aplicando-se os critérios, foram convidadas 34 elegíveis, sendo que três não aceitaram e uma foi excluída por apresentar dificuldades de comunicação causadas por transtorno mental agudo, não havendo desistências. Assim, com amostragem não probabilística, a amostra se constituiu por 30 participantes, representando 42,86% do total. Esse número atendeu à saturação teórica, pois, na análise prévia dos depoimentos, constatou-se que os dados produzidos eram suficientes para compreender o fenômeno e alcançar o objetivo, como se recomenda na abordagem qualitativa^(19)^.

### Coleta dos Dados

Os dados foram produzidos entre março e junho de 2022, por meio de entrevistas individuais realizadas pela pesquisadora principal, mestranda em enfermagem capacitada em reuniões de orientação com a segunda e a terceira autoras e nos encontros regulares de um grupo de pesquisa focado, sobretudo, no desenvolvimento de estudos qualitativos, articulados ou não com a TRS. Em visitas preliminares, ela se apresentou aos gestores das USFs e às equipes multiprofissionais que nelas atuavam, visando facilitar a identificação das pessoas com TB pulmonar e pactuar uma sala para realizar as entrevistas, respeitando a privacidade e assegurando o conforto dos participantes, sem interferir na rotina dos serviços.

À medida que compareceram às unidades, a pesquisadora os abordou individualmente após os atendimentos e os convidou a participar, direcionando à sala reservada aqueles que aceitaram. Nesse ambiente, ocupado somente pela pesquisadora e pelo participante, os objetivos, procedimentos, riscos e benefícios foram apresentados e esclarecidos com linguagem acessível para facilitar o entendimento e obter o aceite formal.

Cada entrevista foi guiada por roteiro semiestruturado, construído pelos autores, com nove perguntas fechadas para caracterizar o perfil sociodemográfico e epidemiológico dos participantes, de acordo com as variáveis idade, sexo, situação conjugal, religião, escolaridade, inserção no mercado de trabalho, renda familiar mensal, unidade de saúde na qual era acompanhado e tipo de entrada (caso novo, recidiva, reingresso após abandono ou transferência), cujas respostas foram registradas manualmente na versão impressa.

Além disso, compunha-se por perguntas abertas para explorar o fenômeno, contemplando aspectos como saberes acerca da humanização da assistência às pessoas com TB e a outros grupos humanos, relacionamento com profissionais que atuavam nas unidades, e experiências de orientação a partir das informações compartilhadas por eles. As respostas a essas perguntas foram audiogravadas em formato MP3, com dispositivo eletrônico, durando, aproximadamente, de 30 a 40 minutos.

O roteiro não foi submetido a um teste piloto, mas foi avaliado por quatro professores doutores que pesquisavam diversos temas nos campos da enfermagem e da saúde coletiva, os quais referendaram o seu conteúdo, possibilitando alcançar o objetivo com entrevistas em profundidade, sem necessitar repeti-las e sem demandar outras técnicas de produção.

### Análise e Tratamento dos Dados

Os dados do perfil sociodemográfico e epidemiológico foram armazenados em planilha do *Microsoft Office Excel* ^®^ (versão 2021) e tratados com estatística descritiva para calcular os números relativos a partir dos números absolutos. As respostas às perguntas abertas foram transcritas integralmente para construir o *corpus* e submetê-lo, em arquivo único, à análise lexical com o *software Analyse Lexicale par Contexte d’un Ensemble de Segments de Texte* (Alceste^®^, versão 2012), que processa dados textuais com métodos estatísticos sofisticados para efetuar o tratamento lexicográfico. Assim, de forma criteriosa, permite interpretar os textos analisados, rastreando os vocabulários (léxicos) utilizados pelos participantes, além da sua ocorrência, coocorrência e associação estatística (*Phi*)^(20)^.

No Alceste^®^, o texto de cada entrevista é denominado Unidade de Contexto Inicial (UCI), e o conjunto das UCIs forma o *corpus*. Esse *software* organiza as palavras em dendrogramas de classes lexicais, de acordo com os seus radicais, e os significados de tais classes são captados nas Unidades de Contexto Elementar (UCEs), que correspondem aos trechos selecionados pelo *software* a partir das UCIs analisadas^(20)^.

Considerou-se, especificamente, a Classificação Hierárquica Descendente, organizada em ordem decrescente, respeitando tanto o índice de associação estatística dos radicais (*Phi*) às classes quanto as suas UCEs correspondentes. Os dados foram interpretados e discutidos com base nas evidências pertinentes da literatura científica sobre o tema e nos preceitos da TRS, apresentados na introdução à luz das ideias de Serge Moscovici^(16)^ e de Denise Jodelet^(17)^, principal teórica da vertente processual.

### Aspectos Éticos

Obedecendo-se aos preceitos éticos da Resolução nº 466/2012 do Conselho Nacional de Saúde, a pesquisa foi autorizada pela SESMA e aprovada por Comitê de Ética em Pesquisa da Universidade do Estado do Pará, sob Parecer nº 5.214.278, emitido em janeiro de 2022. Todos os participantes assinaram o Termo de Consentimento Livre e Esclarecido previamente às entrevistas, declarando o aceite formal e voluntário. Preservou-se o sigilo das suas identidades com a utilização de códigos alfanuméricos formados pela sigla UCI, seguida por hífen e número cardinal, que indica a ordem das entrevistas.

## RESULTADOS

Dos 30 participantes, 22 (73,33%) tinham entre 18 e 49 anos, sendo 19 (63,33%) do sexo masculino. Além disso, 14 (46,67%) eram casados ou viviam em união consensual; 15 (50%) eram evangélicos; 11 (36,67%) referiram ensino fundamental incompleto, e 11 (36,67%), ensino médio completo; 11 (36,67%) estavam desempregados; 19 (63,33%) tinham renda familiar mensal entre um e três salários mínimos; e 26 (86,67%) eram casos novos de TB.

Com o Alceste^®^, o *corpus* foi segmentado em 470 UCEs, compostas por 2.345 palavras distintas, resultando no aproveitamento de 363 UCEs (77,23%), considerando todo o conteúdo das UCIs. Foram geradas cinco classes lexicais, destacando-se as classes 2 e 3, por evidenciarem as RS sobre a humanização da assistência, motivo pelo qual serão aqui apresentadas, com alguns excertos emblemáticos que demonstram os sentidos que lhes caracterizam, possibilitando atender ao objetivo do estudo.

A classe 2 se constituiu por 60 UCEs (16,53%) e 66 palavras analisáveis. Por sua vez, a classe 3 apresentou 76 UCEs (20,94%) e 84 palavras analisáveis. Os radicais, as palavras plenas e os correspondentes valores de *Phi*, atribuídos pelo Alceste^®^, são apresentados no Quadro 1. Para organizar os resultados, os autores conferiram os títulos “A assistência humanizada a partir das relações interpessoais” e “Obstáculos à prática da humanização”, respectivamente para essas classes, tendo em vista seus conteúdos.

**Quadro 1:** Classificação Hierárquica Descendente das classes 2 e 3, com radicais, palavras plenas e seus valores de *Phi* – Belém, PA, Brasil, 2022.

Classe 2			Classe 3		
Radicais	Palavras plenas	*Phi*	Radicais	Palavras plenas	*Phi*
Ouvi	Ouvi	0,33	Atendid	Atendida, atendidas, atendido	0,32
Atendi	Atendimento	0,31	Entreg	Entrega, entregando, entregar, entrego, entregou	0,26
Profission	Profissionais, profissional	0,29	Reclam	Reclamação, reclamam, reclamando, reclamar, reclamavam	0,26
Trat	Trata, tratam, tratar, trataram, tratava, tratou	0,28	Espaço	Espaço	0,25
Acontec	Aconteça, acontece, acontecer, acontecesse, aconteceu	0,26	Fila	Fila, filas	0,24
Paciente	Paciente, pacientes	0,26	Precis	Precisa, precisam, precisamos, precisando, precisar, precisava	0,24
Humanização do cui	Humanização_do_cuidado	0,26	Receb	Recebemos, receber, recebesse, recebo	0,22
Cuidado humanizado	Cuidado_humanizado	0,25	Estrutura	Estrutura	0,22
Pergunt	Pergunta, perguntam, perguntando, perguntar, perguntas	0,23	Acab	Acaba, acabando, acabar, acabei, acabo	0,22
Dão	Dão	0,23	Atend	Atende, atendem, atender, atendeu	0,22
Ouç	Ouça, ouço	0,23	Paciente	Paciente, pacientes	0,22
Comentar	Comentarem, comentários	0,23	Beb	Beber	0,20
Arrogância	Arrogância	0,23	Loc	Locais, local	0,20
Escut	Escutando, escutar, escutou	0,20	Recebid	Recebido	0,19

### Classe 2 – A Assistência Humanizada a Partir das Relações Interpessoais

Os radicais mais significativos dessa classe e as UCEs que lhes deram sentido apontaram que a assistência humanizada se expressa nas relações interpessoais que ocorrem na APS, e a compreensão do fenômeno da humanização se ancorou nas experiências do cotidiano, principalmente por meio do bom trato:

*Nunca ouvi falar em humanização do cuidado! Talvez seja sobre se o atendimento é bom ou não. Acho que um bom atendimento é quando as enfermeiras nos tratam bem, sem ignorância, porque têm alguns lugares que nos tratam com arrogância.* (UCI-1)

*Penso que* [cuidado] *humanizado é o médico tratar bem seu paciente e este concordar com suas palavras.* (UCI-2)

O bom atendimento, traduzido no bom trato, foi entendido por meio de elementos que qualificam atitudes e comportamentos nas relações interpessoais, a partir da postura atenciosa do profissional, das conversações, da empatia e da paciência:

*Os profissionais daqui tratam todos iguais, são muito atenciosos. Durante minhas vindas aqui, o cuidado que recebi sempre foi bom; não tenho por que reclamar.* (UCI-6)

*Ser bem atendido é quando os profissionais conversam com o paciente sem arrogância, e mesmo que o posto* [referindo à unidade de saúde] *não seja de qualidade, eles fazem o que podem para fazer o paciente se sentir bem.* (UCI-23)

Nesse sentido, a escuta qualificada e o interesse pela pessoa com TB foram citados como elementos fundamentais para que se sinta acolhida. Todavia, quando isso não ocorreu em determinadas situações na experiência dos participantes, as relações entre profissional e usuário foram interpretadas a partir de ações discriminatórias, marcadas por notável desinteresse:

*Em outros lugares, os profissionais não dão atenção, apenas anotam no papel e não querem escutar como o paciente se sente. A enfermeira deste posto de saúde me liga, manda mensagem, procura saber como estou, e isso é bom.* (UCI-10)

*Neste posto, nunca me trataram com indiferença, mas, no primeiro* [posto] *que busquei atendimento, sim. Fui com uma assistente social para realizar minha matrícula, e ela não me escutou, não procurou saber onde era mais próximo para eu ser atendida. Me jogou para este posto, com um papel de encaminhamento errado, e ainda me entregou um lenço de papel e mandou colocar mais uma máscara, quando eu já estava com duas.* (UCI-20)

Por esse motivo, o acolhimento dos usuários e a compreensão da sua realidade pelos profissionais ajudaram a caracterizar a assistência humanizada. Ao interpretarem o atendimento de forma humanizada pelo prisma do cuidado oferecido pelo profissional, objetivaram-no como alguém respeitoso, bondoso e que não confunde sua vida pessoal com a vida profissional:

*Para que esse tipo de atendimento aconteça, é necessário um bom profissional, porque há alguns que misturam problemas pessoais com o trabalho, e não conseguem fazer o cuidado humanizado.* (UCI-13)

[...] é *preciso haver profissionais que entendam e compreendam a situação das pessoas que chegam em busca de saúde, porque não são todos que atendem e acolhem bem.* (UCI-18)

### Classe 3 – Obstáculos à Prática da Humanização

Nessa classe, os radicais que indicaram as palavras mais significativas ganharam sentido nas UCEs que revelaram obstáculos à prática da humanização, como as limitações na estrutura física das unidades de saúde e a carência de recursos materiais, que comprometiam o trabalho dos profissionais e, por conseguinte, o atendimento dos usuários:

*O espaço é pequeno para atender à grande quantidade de pacientes. Não tem cadeira para todos e é muito quente.* (UCI-11)

*Precisa melhorar a estrutura física do prédio e os recursos materiais para que os profissionais consigam trabalhar melhor e, assim, os pacientes sejam favorecidos.* (UCI-22)

Os participantes compreendiam que o aperfeiçoamento dos serviços dependia de investimentos dos gestores públicos, incluindo melhorias no espaço físico e a compatibilidade de recursos humanos em relação ao quantitativo de usuários, aproximando-se do conhecimento reificado para elaborar suas RS:

*O que ainda precisa melhorar está relacionado à responsabilidade do município. Por exemplo, colocar mais médicos para atender e melhorar o espaço, que ainda é pequeno, para uma população que aumenta todos os dias.* (UCI-I7)

*Acho que o governo poderia investir mais para melhorar a estrutura física e a segurança do posto, ampliar o espaço e colocar mais profissionais para atender.* (UCI-23)

Considerando a rotina das ações para controlar a TB nessas unidades, afirmaram que a adesão ao tratamento dependia da maneira como eram atendidos pelos profissionais, de modo que, caso se sentissem desrespeitados ou fossem tratados com preconceito, abandonariam o tratamento e o buscariam em outro local, evidenciando fortemente o universo consensual para julgar a viabilidade dessa adesão:

*Se aqui eu percebesse algum comportamento preconceituoso dos profissionais, com certeza não voltaria e desistiria do tratamento ou continuaria em outro lugar.* (UCI-22)

*Se eu recebesse um tratamento ruim, com certeza teria desistido do tratamento aqui, procuraria outro local ou tomaria remédio caseiro.* (UCI-27)

## DISCUSSÃO

Os depoimentos revelaram que os participantes, na tentativa de explicar o fenômeno, representaram a humanização a partir das relações interpessoais, assentadas na ideia de um bom atendimento, e também a partir do que seria o seu oposto, a “desumanização”, a qual foi ancorada nas experiências cotidianas, incluindo os problemas estruturais das unidades de saúde em que eram atendidos, onde deveria ocorrer maior preocupação da gestão pública para efetivar a PNH. Nesse contexto, identificou-se íntima relação entre o conhecimento reificado e o universo consensual.

Evidenciada pelo perfil dos participantes, a pertença grupal configura importante elemento dos estudos baseados na TRS, pois permite entender o contexto a partir do qual expressam suas representações, o que Jodelet denomina como “mundo de vida”^(17)^. Verificou-se, por exemplo, que a faixa etária predominante se aproximou aos resultados do Boletim Epidemiológico de TB, publicado em 2024, que apontou as faixas etárias de 20 a 34 e 35 a 49 anos como majoritárias no sexo masculino, e de 20 a 34 anos, no sexo feminino^(3)^. A prevalência entre homens segue a tendência global de adoecimento por TB^(1,3)^, corroborada também por outros estudos^(13,14)^.

O dado acerca do desemprego indica um cenário preocupante, pois o tratamento é longo, custoso e demanda alimentação adequada. Aos que não possuem renda, pode ser ainda mais desafiador atender às necessidades fisiológicas que advêm da doença e do processo terapêutico, incluindo as necessidades nutricionais, resultando no possível abandono ou na resposta insuficiente do organismo ao tratamento, devido à subnutrição. Em vista disso, no território brasileiro, o Ministério da Saúde tem promovido iniciativas de proteção social às pessoas e às famílias afetadas pela TB, considerando a estreita relação entre pobreza e doença. Assim, realiza ações intersetoriais que envolvem saúde, educação e assistência social, visando ao bem-estar dos acometidos a partir da integralidade da assistência^(6)^.

As ações inerentes à saúde e à educação são desenvolvidas por meio das práticas de atenção realizadas por equipes multiprofissionais. Essas práticas devem articular o cuidado às pessoas com TB com o cuidado àquelas com quem partilham o adoecimento (familiares, cuidadores e acompanhantes), focalizando aspectos como tratamento, prevenção de complicações, reabilitação e reinserção social dos acometidos, mas também busca ativa, avaliação e monitoramento das condições clínicas, prevenção da doença e, se necessário, diagnóstico, tratamento e reabilitação entre os contatos^(2)^.

Dependendo das necessidades biopsicossociais dos acometidos e de seus contatos, isso requer a participação conjunta de tais setores, pois sabe-se que o nível e as condições educacionais influenciam a história natural de doenças socialmente determinadas, como a TB, favorecendo ou reduzindo a disposição e as possibilidades individuais e coletivas para compreender e incorporar práticas de autocuidado e prevenção, tornando-as elementos que ratificam a importância da educação em saúde. Além disso, é por meio da educação que os recursos humanos se qualificam para atuar no controle da TB, razão pela qual ações sistemáticas devem ser propostas e realizadas para estimular a educação continuada e a educação permanente, visando sanar as limitações que advêm do trabalho em saúde ou atenuar seus efeitos^(2)^.

Tecer essa explicação à luz da TRS implica lembrar que as RS estão inseridas em um contexto pré-existente repleto de tradições, valores e crenças que são ancorados ao objeto que se representa, o qual é incorporado às explicações cotidianas do indivíduo e do seu grupo. Tal composição ou troca de saberes/experiências é imprescindível para construir pensamentos assentados no universo consensual e para compartilhá-los com o grupo, oportunizando o pertencimento social^(16)^.

O acesso aos programas sociais de suporte e de transferência de renda, como Aposentadoria para Pessoas de Baixa Renda, Benefício de Prestação Continuada, Programa Bolsa Família, Programa Minha Casa/Minha Vida e Tarifa Social de Energia Elétrica, vigentes no Brasil, pode mitigar as repercussões socioeconômicas da TB no orçamento familiar e fortalecer a qualidade de vida^(21,22)^. Embora não sejam específicos para pessoas com a doença, entende-se que o conhecimento acerca desses programas e a possibilidade de implementá-los são fundamentais para qualificar a assistência, sobretudo às pessoas de baixa renda^(23)^. Visando ao enfrentamento coletivo da TB, isso reforça a articulação que deve existir entre o SUS e o Sistema Único de Assistência Social (SUAS), como reiterado pelo Ministério da Saúde e pelo Ministério da Cidadania, ao publicarem a Instrução Operacional nº 1, de 26 de setembro de 2019^(24)^.

No tocante às RS, ancoradas em um conjunto de categorias do senso comum e no entendimento do processo terapêutico como bom ou ruim, os participantes construíram seus saberes acerca da humanização a partir de oposições, sendo tal entendimento organizado por meio da realidade vivenciada no cuidado recebido. Isso ocorre porque os paradigmas armazenados na memória social estabelecem uma relação positiva ou negativa com o conteúdo que lhe é inerente, o qual é acessado a partir da atividade cognitiva^(16)^.

A palavra “humanização” apresenta difícil compreensão conceitual, mas, quando associada às experiências nos serviços de saúde (dimensão da informação), os participantes demonstraram entender que tratar bem o outro e optar por um relacionamento profícuo (dimensão da atitude) gera comportamentos positivos no contexto social (dimensão da imagem). Assim, identificam-se as três dimensões das RS^(16)^, estruturando o fenômeno da humanização associado ao cuidado assistencial.

Nesse contexto, os participantes materializaram a humanização a partir do bom atendimento que deve ser dispensado pelos profissionais, caracterizando-se por elementos como escuta qualificada, acolhimento, empatia e respeito, amplamente desejados no cotidiano dos serviços em todos os níveis de atenção (primário, secundário/especializado e terciário/hospitalar). Outras pesquisas mostraram resultados semelhantes, quando usuários ancoraram a humanização às boas práticas da equipe de saúde, ansiando por serem tratados com carinho, educação e bom humor, mencionados como ações que identificavam, na sua concepção, um bom atendimento^(25,26)^.

Cognição e afeto se entrelaçam na construção dos saberes e nos processos de aprendizagem, pois o desenvolvimento humano é mediado pela cultura, assim como pelas emoções e interações sociais^(27)^. Portanto, admite-se que as conexões entre aspectos cognitivos, afetivos e sociais influenciam a construção de saberes que produzem sentidos sobre dado objeto^(16,17)^, motivo pelo qual, neste estudo, as RS transitaram dialeticamente entre a desumanização e a humanização da assistência às pessoas com TB, pois diversos elementos da PNH foram citados, ora por sua presença, ora por sua ausência.

Por essa razão, dialogar sem arrogância é condição necessária para que relações horizontais sejam estabelecidas, facilitando a compreensão de informações partilhadas entre pessoas. Isso está contemplado na PNH e, portanto, deve gerar o conjunto de ações conhecido como acolhimento, essencial às relações de cuidado, pois abrange, entre outros aspectos, o compartilhamento de informações e o vínculo humano para orientar decisões pertinentes e oportunas^(8)^. Da mesma forma, outros estudos apontam relações acolhedoras como expressão humanizada e que facilita a criação de vínculos e do sentimento de confiança, ajudando a solucionar problemas cotidianos^(8,28)^.

Foi sob esse entendimento que os participantes objetivaram o profissional de saúde como alguém respeitoso, bondoso e que não confundia sua vida pessoal com a vida trilhada no seu ofício, na perspectiva de ser alguém que acolhia e era capaz de dirimir os anseios dos usuários. A modificação do abstrato, da ideia-conceito, para o modelo figurativo se configura como parte da objetivação, e esse modelo tem como função apresentar um ponto comum entre os diferentes saberes, traduzir a representação real do objeto e associar os elementos em uma dinâmica que é própria dele^(16)^.

O longo período de tratamento da TB e o acompanhamento na APS possibilitam criar vínculos horizontalizados, mas é fundamental que profissionais e usuários se engajem para fortalecer suas relações interpessoais, as quais devem se fundamentar em cinco pilares: cordialidade; ética; empatia; assertividade; e autoconhecimento. O pilar da cordialidade diz respeito ao fato de alguém ser gentil e solícito, enquanto o da ética se refere ao senso de justiça, princípio e disciplina, sendo ambos necessários para aprimorar o cuidado, tornando-o resolutivo diante dos problemas de saúde, neste caso, da TB^(29)^.

O pilar da empatia é inerente ao ato de se colocar no lugar do outro, dispondo-se a ouvi-lo, contexto em que os resultados desta pesquisa mostraram que a escuta qualificada é um componente essencial das práticas assistenciais^(29)^. A ação de ouvir o outro pode ocorrer em vários momentos, como no acolhimento, que propicia o diálogo oportuno entre profissional e usuário, adequado às suas necessidades, possibilitando a avaliação de vulnerabilidades, gravidade e riscos, de modo a favorecer o compromisso e o vínculo entre esses atores. Assim, é possível ampliar a efetividade das práticas de saúde, contribuindo para a clínica ampliada, por favorecer decisões compartilhadas e comprometidas com a autonomia dos usuários do SUS^(5)^.

Nessa perspectiva, chamou atenção a representação da assistência humanizada por meio de duas ações cognitivas humanas: “compreender” e “entender”, mencionadas por UCI-18 na classe 2. Compreender é um processo contínuo; por isso, ao tratar desse conceito, admite-se não haver algo definido e imutável, pois o que existe é uma compreensibilidade em constante transformação. Entender, por sua vez, consiste em ouvir, perceber e assimilar o propósito de algo^(30)^.

Dessa maneira, compreender implica uma ação muito envolvente, permeada de compromisso com o conhecimento acerca dos aspectos mais profundos do sujeito, enquanto entender configura um processo mais superficial e derivado da palavra “compreender”. Interpretar a assistência humanizada por meio de ambas as palavras evidencia que esse tipo de assistência se reveste por características próprias da interação, e ainda que a estrutura institucional seja sofisticada e o ambiente seja acolhedor, nada supera a presença do profissional, pois somente um ser humano é capaz de compreender o outro^(30)^. Logo, quando os usuários se esforçam cognitivamente para representar a assistência humanizada à luz de suas experiências, a compreensão se eleva como elemento de destaque e que traduz a relação humana, tão necessária ao cuidado^(31)^.

Para compreender o outro, é preciso estabelecer contato para ajudá-lo a superar as adversidades que podem interferir no cuidado de si, no tratamento e na cura. Mudar perspectivas sobre hábitos de vida que adoecem ou que tornam a cura mais lenta requer, muitas vezes, a necessária (re)classificação de ações cotidianas, inclusive nas relações e inserções sociais, exigindo que o profissional estabeleça vínculos de confiança com o usuário para que, na construção de um diálogo horizontal, o cuidado se efetive^(5,30)^.

O pilar da assertividade fortalece as relações interpessoais, quando trata das interações entre profissional e usuário, baseadas na comunicação clara, direta e respeitosa. Por sua vez, o pilar do autoconhecimento permite ao profissional compreender sua personalidade para evitar ou amenizar conflitos^(29)^, como indicado nos resultados deste estudo, quando os participantes reconheceram que é importante separar os aspectos da vida pessoal em relação à vida profissional.

Tratando-se da humanização, é oportuno ressaltar que, sem iniciativas adequadas de educação permanente, não há qualidade satisfatória das práticas assistenciais^(5)^. Portanto, referindo-se à necessidade de os problemas pessoais não interferirem no bom atendimento, os participantes denotaram que os profissionais deveriam ter não apenas capacidade técnico-científica, mas também competências e habilidades relacionais para, inclusive, combater ações desumanizadas, que repercutiam na capacidade de compreender o usuário.

Outro aspecto importante diz respeito aos elementos do ambiente, pois influenciam fortemente a assistência e, por consequência, o tratamento e a cura. Analisando a PNH, observa-se que o ambiente é um instrumento humanizador, visto que deve ajudar a promover conforto, segurança e bem-estar, além de ser um elemento que integra a equipe multiprofissional, os usuários e os seus familiares^(25)^.

Considerando as especificidades da TB, o Ministério da Saúde recomenda que, na APS, haja correto planejamento arquitetônico das unidades de saúde para evitar a disseminação do *Mycobacterium tuberculosis* e de outros agentes patogênicos transmitidos por via aérea, valorizando itens como iluminação e ventilação adequadas, pois, no caso da TB, os bacilos expelidos por pessoas com a doença podem se dispersar rapidamente^(2)^. Assim, é fundamental que haja espaços cujas características garantam conforto a essas pessoas e segurança sanitária aos outros grupos que ali frequentarem, propiciando a quebra da cadeia de transmissão.

O município de Belém se caracteriza por um clima quente e úmido, com períodos de pouca ou muita chuva^(32)^. Por isso, espaços pequenos em unidades da APS, sobretudo no cotidiano das atividades para controlar a TB, podem interferir significativamente na saúde humana, tendo em vista que ambientes fechados propiciam a dispersão de bacilos^(2)^ e a circulação de partículas e pequenos organismos, como poeira e ácaros^(33)^.

Segundo a PNH, o conceito de ambiência necessariamente incorpora espaços acolhedores, confortáveis e que garantam a privacidade dos grupos humanos, mas que também favoreçam, sempre que necessário, mudanças no processo de trabalho, promovendo encontros pautados no diálogo^(5)^. Assim, esses espaços devem oferecer aos profissionais e usuários ambientação adequada e interativa.

Contrastando com essas informações, os depoimentos revelaram a imagem de um espaço pequeno e que dispunha de poucas cadeiras, em quantidade insuficiente para atender à demanda dos que buscavam atendimento. Essa realidade dificultava o processo de trabalho multiprofissional e as relações afetivas, reduzindo o bem-estar dos usuários no contexto assistencial.

Faz-se tal ponderação porque, além das representações que circulam sobre a TB, há os desafios enfrentados pelos sujeitos. Esses desafios se apresentam de maneira cumulativa e resultam em aspectos negativos e/ou positivos diante das experiências em torno da doença, permitindo construir novos olhares e significados que serão benéficos ou deletérios, fortalecedores ou limitantes, necessários ou desnecessários para o cotidiano e para o enfrentamento à TB, dependendo da natureza dos seus conteúdos^(34)^.

Assim, os participantes apontaram a gestão pública como responsável por proporcionar melhorias nos serviços, mediante investimentos destinados efetivamente à APS. Nesse sentido, reconheceram que as equipes das unidades operacionalizavam ações de saúde, mas cabia aos gestores prover os recursos necessários. Por esse motivo, entende-se que a ausência de recursos básicos para qualificar os serviços foi caracterizada como condição desumana, pois repercutia na saúde tanto do profissional quanto do usuário.

Isso significa que a desumanização pode ser evidenciada não somente por meio de atitudes e comportamentos, mas também pela carência de estrutura e recursos, que obstaculizam a adesão terapêutica entre pessoas com TB^(28)^. Essa compreensão ajuda a explicar a decisão dos participantes em buscar outros serviços ou optar por outras formas de tratamento, caso o atendimento recebido nas unidades fosse desrespeitoso ou apresentasse indícios de preconceito.

Nessa perspectiva, a interlocução entre o conhecimento reificado e o universo consensual possibilita a dinamicidade das RS, contexto no qual as mudanças de concepção e a união dos saberes que advêm de ambas as lógicas de pensamento são fundamentais para (re)construir essas RS. Também possibilita compreender como o conhecimento científico é apropriado por pessoas no discurso social e se torna conteúdo comum do saber popular, fornecendo elementos para caracterizar os modos como grupos sociais pensam e agem diante de um objeto ou contexto com importância subjetiva^(35)^.

Tal resultado salienta que as RS sobre a assistência humanizada, no contexto da TB, se sustentam na ideia de um bom atendimento como referência para manter o usuário em acompanhamento. Esse é um elemento importante, pois demonstra que os investimentos adequados na formação técnico-científica e relacional dos profissionais são importantes para efetivar o tratamento^(2)^.

Para a PNH, humanizar a assistência significa valorizar os diferentes sujeitos implicados no processo de produção da saúde (usuários, trabalhadores e gestores), incluindo valores como autonomia e protagonismo, corresponsabilidade, estabelecimento de vínculos solidários, e participação coletiva no processo de gestão^(31)^. Um sistema de saúde que funcione adequadamente, com gestão participativa, financiamento, força de trabalho e recursos tecnológicos, estimula o progresso da APS^(36)^.

Portanto, é necessário qualificar a PNH e ampliar os modos de fazer dos profissionais que nela atuam, construindo alternativas que valorizem diferenças, singularidades e práticas humanizadas, as quais devem se sustentar em atitudes baseadas no respeito mútuo, mas também em ações com foco no comprometimento coletivo. Ressalta-se que, no âmbito da saúde, mudanças estruturais e socioculturais, direcionadas à qualidade e à humanização da assistência, podem se tornar importantes elementos no processo de ampliação do campo simbólico das RS sobre a humanização^(37)^.

As limitações deste estudo correspondem à sua realização em município específico de um estado brasileiro, motivo pelo qual a abrangência dos seus resultados se restringe a cenários com características semelhantes ou aproximadas, tornando necessário desenvolver outros estudos de RS sobre o tema, de modo a contemplar os três níveis de atenção. Assim, é preciso compreendê-lo em outros espaços sociais, ajudando a construir e disseminar reflexões pautadas na valorização igualitária do conhecimento reificado e do universo consensual, lógicas de pensamento que possibilitam identificar as RS.

Perante seus resultados, este estudo contribui com as áreas de enfermagem e saúde coletiva, na medida em que pode subsidiar o diálogo profícuo entre estudantes, profissionais, gestores e autoridades públicas para qualificar os serviços de saúde e propor novas estratégias que fortaleçam a humanização no cotidiano da assistência às pessoas com TB.

Faz-se tal ponderação considerando que, no contexto prático, esses atores sociais podem ser estimulados a pensar criticamente sobre os fatores que obstaculizam a prática da humanização em serviços da APS. Exercício reflexivo consistente pode orientá-los na proposição de medidas que repercutam sobre os diferentes elementos que envolvem a humanização, entre os quais destacam-se as características estruturais das unidades de saúde, os processos de trabalho multiprofissional e as relações humanas que neles são tecidas com os usuários, como apontado nos depoimentos. Para que sejam efetivas e fortaleçam a humanização, tais medidas devem considerar os desafios administrativos, materiais, operacionais, políticos e socioculturais inerentes a esses serviços, visando transformar as suas realidades em algum grau.

## CONCLUSÃO

Evidenciou-se que a humanização da assistência foi ancorada nas relações interpessoais, estabelecidas entre profissionais de saúde e usuários, de modo que fossem sustentadas pelo sentimento de confiança e pelo bom trato, característicos de um atendimento qualificado. Todavia, na realidade dos participantes, é possível inferir que não foi concretizada, devido às limitações na estrutura física das unidades de saúde onde eram atendidos e à carência de recursos materiais, reconhecidos como problemas, inerentes à gestão pública, que precisavam ser superados para fortalecer as ações de controle da TB na APS.

Nesse sentido, as relações interpessoais configuraram o esteio da humanização, sendo por meio delas que se viabilizavam a compreensão mútua sobre a doença, o processo terapêutico e as adversidades que poderiam ocorrer nesse contexto, favorecendo a adesão ao tratamento. Assim, conhecer os elementos que constituíam ou influenciavam as RS de pessoas com TB acerca do tema demonstrou a importância das relações humanas nas atividades assistenciais, sobretudo perante os desafios do trabalho multiprofissional na APS, incentivando a construção e o fortalecimento de vínculo entre equipes e usuários.

Diante das RS aqui desveladas, entende-se que novas pesquisas podem ajudar a compreender outros aspectos psicossociais em torno da humanização, resultando em contribuições para a assistência, a gestão dos serviços, o ensino e a pesquisa no campo da saúde. Essas esferas de poder e atuação sócio-política são inerentes a diversos profissionais, incluindo o enfermeiro, cujas responsabilidades ética, social e técnica guiam a identificação das necessidades dos grupos humanos para aplicar intervenções que transformem, em algum grau, a realidade que vivenciam, possibilitando incorporar práticas de cuidado individualizado.

## Data Availability

Os dados que dão suporte a este estudo podem ser disponibilizados mediante solicitação ao autor correspondente.

# Translation: Humanización de la asistencia en la Atención Primaria de Salud: representaciones sociales de personas con tuberculosis pulmonar*

**DOI:** 10.1590/1980-220X-REEUSP-2025-0008es

**Authors:** Mayara Del Aguilal Pacheco, Iací Proença Palmeira, Márcia de Assunção Ferreira, Aloma Sena Soares, Erlon Gabriel Rego de Andrade, Alexandre Aguiar Pereira, Ivaneide Leal Ataíde Rodrigues, Widson Davi Vaz de Matos

**Affiliations:** 1 Universidade Federal do Rio de Janeiro, Escola de Enfermagem Anna Nery, Programa de Pós-Graduação em Enfermagem, Rio de Janeiro, RJ, Brasil.; 2 Universidade do Estado do Pará, Escola de Enfermagem Magalhães Barata, Programa de Pós-Graduação em Enfermagem, Belém, PA, Brasil.

**Keywords:** Humanización de la Atención, Atención Primaria de Salud, Tuberculosis Pulmonar, Representación Social, Psicología Social

## Abstract

**Objetivo:**

Analizar las representaciones sociales de las personas con tuberculosis pulmonar respecto a la humanización de la atención en Atención Primaria de Salud.

**Método:**

Investigación descriptiva con enfoque cualitativo, basada en el aspecto procedimental de la Teoría de las Representaciones Sociales. Se llevó a cabo en cuatro Unidades de Salud Familiar de Belém, Pará, Brasil, con 30 personas en tratamiento por tuberculosis pulmonar. Entre marzo y junio de 2022, se realizaron entrevistas individuales semiestructuradas, cuyo *corpus* se sometió al *software Analyse Lexicale par Contexte d’un Ensemble de Segments de Texte* (versión 2012).

**Resultados:**

El *corpus* se dividió en 470 Unidades de Contexto Elemental, de las cuales se utilizaron 363 (77,23%). Se generaron cinco clases léxicas, destacando las clases 2 y 3 por resaltar las representaciones sociales del tema, un contexto en el que la humanización de la atención se sustentaba en un sentido de confianza y buen trato, a pesar de las limitaciones en la estructura física y los recursos materiales de las unidades de salud.

**Conclusión:**

Las relaciones humanas son importantes en las actividades de salud, especialmente delante de los desafíos del trabajo multidisciplinario en la Atención Primaria de Salud, fomentando la construcción y fortalecimiento de vínculos entre equipos y usuarios.

## INTRODUCCIÓN

Enfermedad milenaria, conocida popularmente como tisis o peste blanca, la tuberculosis (TB) se caracteriza por ser infecciosa, contagiosa, prevenible y curable. Su etiología está asociada a la infección por bacilos de la especie *Mycobacterium tuberculosis* que, al ser expulsados por una persona bacilífera al estornudar, hablar o toser, pueden contaminar de diez a quince individuos en una comunidad. Se manifiesta en dos formas clínicas, denominadas TB pulmonar, cuyos órganos afectados son los pulmones, y TB extrapulmonar, que afecta a otros órganos y sistemas^(1,2)^.

En 2023, se estimó que 10,8 millones de personas enfermaron y 1,25 millones murieron de TB en todo el mundo^(1)^. En Brasil, se notificaron 80.012 casos nuevos y, en 2022, 5.845 muertes, lo que resultó en coeficientes de incidencia y mortalidad iguales a 37,3 casos/100 mil habitantes y 2,8 muertes/100 mil habitantes. En el estado de Pará, considerando los mismos años, se registraron 4.293 casos nuevos y 341 muertes, ocupando el primer lugar en número absoluto de casos nuevos y muertes por la enfermedad en la región norte del país, con coeficientes de 48,8 casos/100 mil habitantes y 3,9 muertes/100 mil habitantes. Por su parte, en el municipio de Belém se registraron 1.360 casos nuevos y 135 muertes, con coeficientes de 90,7 casos/100 mil habitantes y 9,0 muertes/100 mil habitantes^(3)^.

Se sabe que los vínculos entre los profesionales de la salud y los usuarios son fundamentales para mejorar la calidad de la atención y promover el control efectivo de la enfermedad como problema de salud pública^(4)^. Corroborando esto, la Política Nacional de Humanización (PNH), instituida por el Ministerio de Salud en 2003, tiene como objetivo mejorar la atención en el Sistema Único de Salud (SUS) para proporcionar acciones más acogedoras y participativas, por lo que presenta, como uno de sus principios, el protagonismo de los usuarios y los trabajadores. Para lograr un enfoque integral y humanizado, propone un modelo de atención que valora la escucha cualificada, la corresponsabilidad y la ampliación del diálogo entre profesionales y usuarios en el proceso terapéutico, respetando la dignidad humana y el acceso equitativo a los servicios de salud^(5)^.

En la atención a las personas con TB, la PNH desempeña un papel crucial al fomentar prácticas que superen la fragmentación de la atención y garanticen un seguimiento continuo, esencial para que los afectados se adhieran al uso de medicamentos antimicrobianos durante un largo periodo. La implementación exitosa de la PNH requiere estructuras de gestión participativa que garanticen, entre otros elementos, la participación de los usuarios en la toma de decisiones clínicas, su protagonismo para exigir que los profesionales tengan actitudes y comportamientos humanizados, la capacitación continua de los profesionales y el diálogo resolutivo entre los participantes^(5)^. Los enfoques que valoran el diálogo, los vínculos y la integralidad propician acciones de cuidado personalizadas y eficaces, reduciendo los estigmas y otras barreras socioculturales que, con frecuencia, limitan el éxito terapéutico entre las personas con TB^(6,7)^.

La humanización también se expresa como un fenómeno relacional, con implicaciones para desarrollar buenas relaciones en el entorno laboral y fortalecer los vínculos entre profesionales y usuarios, favoreciendo la prestación de cuidados centrados en las especificidades de los afectados, que requieren una visión holística para que se atiendan sus necesidades biopsicosociales^(8)^. En la vida cotidiana de las personas con TB, atendidas en unidades de Atención Primaria de Salud (APS), las acciones de cuidado de calidad son posibles cuando existe una buena relación interpersonal entre ellas y los profesionales durante el proceso terapéutico, lo que culmina en la construcción o el fortalecimiento del sentimiento de confianza por parte del usuario(^(9,10)^.

Sin embargo, aún se evidencian el miedo y otros afectos negativos entre ciertos profesionales al saber que el usuario tiene TB, reactivando la memoria social del estigma y el prejuicio que los aleja de los afectados, comprometiendo los vínculos^(11)^. Esto se debe a que estos profesionales manifiestan representaciones erróneas sobre la forma de transmitir la TB, lo que indica una posible falta de información cualificada y capaz de influir en sus acciones en el seguimiento terapéutico de los afectados^(12)^.

También se entiende que la falta de información puede generar sentimientos desagradables entre las personas con TB, alimentando representaciones negativas que provienen de la estigmatización y la exclusión social, considerando que la enfermedad se relaciona con creencias y tabúes envueltos en afectos, como el miedo y la tristeza ante el diagnóstico y el tratamiento^(9)^. Así, la ausencia o la falta de cuidados compasivos, empáticos y receptivos a las necesidades de los afectados contribuye al rendimiento insuficiente de las relaciones interpersonales o incluso a la falta de adherencia al tratamiento^(13,14)^.

Las limitaciones de la humanización en la APS deben tratarse como un problema solucionable, cuya resolución es necesaria para el buen funcionamiento del SUS^(8)^. En este contexto, es fundamental asociar los conocimientos técnico-científicos a las acciones humanizadas de los profesionales para el manejo y control adecuados de la TB. Esta consideración se hace teniendo en cuenta que la APS constituye el primer nivel de atención recomendado para atender a las personas con TB, escenario en el que es posible movilizar conocimientos, afectos y comportamientos que acerquen o alejen a los afectados en relación con los servicios de la APS, con repercusiones en los ámbitos individual y colectivo^(15)^.

Las investigaciones sobre el tema apuntan a la Teoría de las Representaciones Sociales (TRS), elaborada por el psicólogo Serge Moscovici, que se centra en las representaciones sociales (RS) como formas de conocimiento particular que organizan diversos saberes en el campo cognitivo y se originan a partir de las comunicaciones y las relaciones en grupo. Las RS están formadas por dos procesos cognitivos basados en la memoria: el anclaje, que integra un objeto social nuevo o inusitado a las construcciones mentales previas; y la objetivación, que materializa ese objeto con imágenes o símbolos de la vida cotidiana sociocultural. Presentan tres dimensiones: la información, que implica la construcción y estructuración de conocimientos procedentes de las interacciones y comunicaciones; la actitud, que se refiere al juicio o posicionamiento del individuo frente al objeto; y el campo representacional o dimensión de la imagen, que se relaciona con otros componentes asociados al objeto, siguiendo la lógica de un modelo social, en el que los elementos de las RS se ordenan y jerarquizan^(16)^.

En este contexto, dos lógicas de pensamiento organizan y expresan las RS: el universo consensual, definido por el conjunto de conocimientos del sentido común; y el conocimiento reificado, conjunto de conocimientos técnicos originados en la ciencia. Al estudiar las RS, es posible comprender cómo y por qué las personas saben, piensan y actúan de cierta manera ante los fenómenos sociales que circulan en su vida cotidiana, lo cual es especialmente relevante cuando se trata de la humanización de la asistencia en el control de la TB.

Las prácticas que corresponden a la atención humanizada se caracterizan por elementos como la escucha sensible y la comunicación de calidad, y promueven vínculos significativos entre profesionales y usuarios, lo que repercute en su forma de actuar y en la adhesión o no a las recomendaciones de salud^(5,8)^. Estas prácticas se construyen desde una perspectiva psicosociológica, marcada por la interacción entre la persona, el profesional y el entorno que los rodea, acercándose a la TRS al mostrar que los conocimientos sobre un objeto están íntimamente relacionados con las acciones de los sujetos^(15,17)^.

La APS es un escenario propicio para construir, difundir y fortalecer las RS, ya que las interacciones moldean las acciones cotidianas^(15,17)^. Por lo tanto, conocer las RS de las personas con TB sobre la humanización, en el primer nivel de atención, puede revelar cómo el pensamiento social influye en los comportamientos de acuerdo con los significados atribuidos durante el proceso terapéutico.

Teniendo en cuenta la relevancia del tema, se formuló la pregunta orientadora: ¿cómo representan las personas con TB pulmonar la humanización de la asistencia en la APS? Para responderla, este estudio tuvo como objetivo analizar las representaciones sociales de las personas con tuberculosis pulmonar sobre la humanización de la asistencia en la Atención Primaria de Salud.

## MÉTODO

### Tipo de Estudio

Investigación descriptiva, con enfoque cualitativo, basada en la vertiente procesual de la TRS. Se optó por esta teoría considerando que permite investigar cómo los sujetos construyen explicaciones sobre un determinado objeto social y lo interpretan a través del lenguaje y las relaciones entre el objeto y las condiciones sociohistóricas y culturales, lo que permite comprender las acciones adoptadas en la vida cotidiana^(16,17)^ Se siguieron las recomendaciones de la guía *COnsolidated criteria for REporting Qualitative research* (COREQ)^(18)^.

### Lugares

Se llevó a cabo en cuatro Unidades de Salud Familiar (USF) ubicadas en el Distrito Administrativo de Sacramenta (DASAC), uno de los ocho distritos administrativos del municipio de Belém, estado de Pará, Brasil. En un estudio previo, realizado en la Secretaría Municipal de Salud de Belém (SESMA), se verificó que, en 2021, el DASAC presentó un gran número de casos registrados de tuberculosis pulmonar: alrededor de 126 (40,13%) del total de 314 registrados en el municipio. En ese mismo año, las unidades seleccionadas presentaron el mayor número de casos en seguimiento en ese distrito, lo que justificó su elección.

### Población y Criterios de Selección

Para seleccionar a los participantes, los criterios de inclusión fueron ser mayor de 18 años, llevar al menos dos meses de tratamiento para la TB pulmonar y estar registrado en una de las USF seleccionadas. La delimitación del período de dos meses consideró el entorno y la convivencia del participante con las rutinas de los servicios de salud y el tratamiento de la TB, criterios fundamentales para captar las RS sobre la humanización de la asistencia, ya que configuran la aplicabilidad sociocultural por la inmersión del objeto en la vida cotidiana del sujeto^(16)^. Los criterios de exclusión fueron las personas con dificultades de comunicación detectadas por la investigadora principal, ya que estas dificultades podrían limitar la participación en las entrevistas.

La población estuvo compuesta por 70 personas en seguimiento en las cuatro USF. Aplicando los criterios, se invitó a 34 personas elegibles, de las cuales tres no aceptaron y una fue excluida por presentar dificultades de comunicación causadas por un trastorno mental agudo, sin que hubiera desistimientos. Así, con un muestreo no probabilístico, la muestra estuvo compuesta por 30 participantes, lo que representa el 42,86% del total. Este número cumplió con la saturación teórica, ya que, en el análisis previo de los testimonios, se constató que los datos producidos eran suficientes para comprender el fenómeno y alcanzar el objetivo, tal y como se recomienda en el enfoque cualitativo^(19)^.

### Recopilación de Datos

Los datos se produjeron entre marzo y junio de 2022, mediante entrevistas individuales realizadas por la investigadora principal, estudiante de maestría en enfermería capacitada en reuniones de orientación con la segunda y tercera autoras y en las reuniones regulares de un grupo de investigación centrado, sobre todo, en el desarrollo de estudios cualitativos, articulados o no con la TRS. En visitas preliminares, se presentó a los administradores de las USF y a los equipos multidisciplinarios que trabajaban en ellas, con el fin de facilitar la identificación de las personas con tuberculosis pulmonar y acordar una sala para realizar las entrevistas, respetando la privacidad y garantizando la comodidad de los participantes, sin interferir en la rutina de los servicios.

A medida que acudían a las unidades, la investigadora se dirigió a ellos individualmente después de las consultas y los invitó a participar, dirigiendo a la sala reservada a aquellos que aceptaron. En ese entorno, ocupado únicamente por la investigadora y el participante, se presentaron y aclararon los objetivos, procedimientos, riesgos y beneficios con un lenguaje accesible para facilitar la comprensión y obtener la aceptación formal.

Cada entrevista se guió por un guion semiestructurado, elaborado por los autores, con nueve preguntas cerradas para caracterizar el perfil sociodemográfico y epidemiológico de los participantes, de acuerdo con las variables de edad, sexo, situación conyugal, religión, escolaridad, inserción en el mercado laboral, ingreso familiar mensual, unidad de salud en la que se les atendía y tipo de ingreso (caso nuevo, recaída, reingreso tras abandono o transferencia), cuyas respuestas se registraron manualmente en la versión impresa.

Además, se componía de preguntas abiertas para explorar el fenómeno, contemplando aspectos como los conocimientos sobre la humanización de la atención a las personas con TB y otros grupos humanos, la relación con los profesionales que trabajaban en las unidades y las experiencias de orientación a partir de la información compartida por ellos. Las respuestas a estas preguntas se grabaron en formato MP3 con un dispositivo electrónico, con una duración aproximada de 30 a 40 minutos.

El guion no se sometió a una prueba piloto, pero fue evaluado por cuatro profesores doctores que investigaban diversos temas en los campos de la enfermería y la salud colectiva, quienes refrendaron su contenido, lo que permitió alcanzar el objetivo con entrevistas en profundidad, sin necesidad de repetirlas y sin requerir otras técnicas de producción.

### Análisis y Tratamiento de los Datos

Los datos del perfil sociodemográfico y epidemiológico se almacenaron en una hoja de cálculo de *Microsoft Office Excel* ^®^ (versión 2021) y se trataron con estadística descriptiva para calcular los números relativos a partir de los números absolutos. Las respuestas a las preguntas abiertas se transcribieron íntegramente para construir el *corpus* y someterlo, en un único archivo, al análisis léxico con el *software Analyse Lexicale par Contexte d’un Ensemble de Segments de Texte* (Alceste^®^, versión 2012), que procesa datos textuales con métodos estadísticos sofisticados para realizar el tratamiento lexicográfico. De este modo, permite interpretar de forma criteriosa los textos analizados, rastreando el vocabulario (léxico) utilizado por los participantes, además de su ocurrencia, coocurrencia y asociación estadística (*Phi*)^(20)^.

En Alceste^®^, el texto de cada entrevista se denomina Unidad de Contexto Inicial (UCI), y el conjunto de las UCI forma el *corpus*. Este software organiza las palabras en dendrogramas de clases léxicas, de acuerdo con sus radicales, y los significados de dichas clases se captan en las Unidades de Contexto Elemental (UCE), que corresponden a los fragmentos seleccionados por el software a partir de las UCI analizadas^(20)^.

Se consideró, específicamente, la Clasificación Jerárquica Descendente, organizada en orden decreciente, respetando tanto el índice de asociación estadística de las raíces (*Phi*) a las clases como sus UCE correspondientes. Los datos se interpretaron y discutieron basándose en las evidencias pertinentes de la literatura científica sobre el tema y en los preceptos de la TRS, presentados en la introducción a la luz de las ideas de Serge Moscovici^(16)^ y Denise Jodelet^(17)^, principal teórica de la vertiente procesual.

### Aspectos Éticos

De conformidad con los preceptos éticos de la Resolución n.º 466/2012 del Consejo Nacional de Salud, la investigación fue autorizada por la SESMA y aprobada por el Comité de Ética en Investigación de la Universidad Estatal de Pará, mediante el Dictamen n.º 5.214.278, emitido en enero de 2022.

Todos los participantes firmaron el Término de Consentimiento Libre e Informado antes de las entrevistas, declarando su aceptación formal y voluntaria. Se preservó el secreto de sus identidades mediante el uso de códigos alfanuméricos formados por la sigla UCI, seguida de un guión y un número cardinal, que indica el orden de las entrevistas.

## RESULTADOS

De los 30 participantes, 22 (73,33%) tenían entre 18 y 49 años, siendo 19 (63,33%) del sexo masculino. Además, 14 (46,67%) estaban casados o vivían en unión libre; 15 (50%) eran evangélicos; 11 (36,67%) declararon no haber completado la educación primaria y 11 (36,67%) habían completado la educación secundaria; 11 (36,67%) estaban desempleados; 19 (63,33%) tenían ingresos familiares mensuales de entre uno y tres salarios mínimos; y 26 (86,67%) eran casos nuevos de TB.

Con Alceste^®^, el *corpus* se segmentó en 470 UCEs, compuestas por 2345 palabras distintas, lo que dio como resultado el aprovechamiento de 363 UCEs (77,23%), considerando todo el contenido de las UCIs. Se generaron cinco clases léxicas, destacando las clases 2 y 3, por evidenciar las RS sobre la humanización de la asistencia, motivo por el cual se presentarán aquí, con algunos extractos emblemáticos que demuestran los significados que las caracterizan, lo que permite cumplir con el objetivo del estudio.

La clase 2 se compuso de 60 UCEs (16,53%) y 66 palabras analizables. Por su parte, la clase 3 presentó 76 UCEs (20,94%) y 84 palabras analizables. Los radicales, las palabras completas y los valores correspondientes de *Phi*, atribuidos por Alceste^®^, se presentan en la Cuadro 1. Para organizar los resultados, los autores asignaron los títulos «La atención humanizada a partir de las relaciones interpersonales» y «Obstáculos a la práctica de la humanización», respectivamente, a estas clases, teniendo en cuenta sus contenidos.

**Cuadro 1:** Clasificación jerárquica descendente de las clases 2 y 3, con radicales, palabras completas y sus valores de *Phi* – Belém, PA, Brasil, 2022.

Clase 2			Clase 3		
Radicales	Palabras plenas	*Phi*	Radicales	Palabras plenas	*Phi*
Oí	Oí	0,33	Atendid	Atendida, atendidas, atendido	0,32
Atendi	Atendimiento	0,31	Entreg	Entrega, entregando, entregar, entrego, entregó	0,26
Profesión	Profesionales, Profesional	0,29	Reclam	Reclamación, reclaman, reclamando, reclamar, reclamaban	0,26
Trat	Trata, tratan, tratar, trataron, trataba, trató	0,28	Espacio	Espacio	0,25
Acontec	Acontece, acontecer, aconteciese, aconteció	0,26	Fila	Fila, filas	0,24
Paciente	Paciente, pacientes	0,26	Precis	Precisa, precisan, precisamos, precisando, precisar, precisaba	0,24
Humanización del cui	Humanización_del_cuidado	0,26	Receb	Recibimos, recibir, recibiese, recibo	0,22
Cuidado humanizado	Cuidado_humanizado	0,25	Estructura	Estructura	0,22
Pergunt	Pregunta, preguntan, preguntando, preguntar, preguntas	0,23	Acab	Acaba, acabando, acabar, acabé acabo	0,22
Dan	Dan	0,23	Atend	Atiende, atienden, atender, atendió	0,22
Oig	Oiga, oigo	0,23	Paciente	Paciente, pacientes	0,22
Comentar	Comentaran, comentarios	0,23	Beb	Beber	0,20
Arrogancia	Arrogancia	0,23	Loc	Locales, local	0,20
Escut	Escuchando, escuchar, escuchó	0,20	Recibid	Recibido	0,19

### Clase 2 – La Atención Humanizada a Partir de las Relaciones Interpersonales

Los radicales más significativos de esta clase y las UCEs que les dieron sentido señalaron que la atención humanizada se expresa en las relaciones interpersonales que se dan en la APS, y la comprensión del fenómeno de la humanización se basó en las experiencias cotidianas, principalmente a través del buen trato:

¡Nunca *había oído hablar de la humanización de la atención! Quizás se trate de si la atención es buena o no. Creo que una buena atención es cuando las enfermeras nos tratan bien, sin ignorancia, porque hay algunos lugares donde nos tratan con arrogancia.* (UCI-1)

*Creo que* [la atención] *humanizada es que el* médico trate *bien a su paciente y este esté de acuerdo con sus palabras.* (UCI-2)

La buena atención, traducida en un buen trato, se entendió a través de elementos que califican actitudes y comportamientos en las relaciones interpersonales, a partir de la actitud atenta del profesional, las conversaciones, la empatía y la paciencia:

*Los profesionales de aquí tratan a todos por igual, son muy atentos. Durante mis visitas aquí, la atención que he recibido siempre ha sido buena; no tengo nada de qué quejarme.* (UCI-6)

*Recibir una buena atención es cuando los profesionales hablan con el paciente sin arrogancia, e incluso si el centro* [refiriéndose a la unidad de salud] *no es de calidad, hacen lo que pueden para que el paciente se sienta bien.* (UCI-23)

En este sentido, la escucha cualificada y el interés por la persona con TB se citaron como elementos fundamentales para que se sienta acogida. Sin embargo, cuando esto no ocurrió en determinadas situaciones en la experiencia de los participantes, las relaciones entre el profesional y el usuario se interpretaron a partir de acciones discriminatorias, marcadas por un notable desinterés:

*En otros lugares, los profesionales no prestan atención, solo anotan en un papel y no quieren escuchar cómo se siente el paciente. La enfermera de este centro de salud me llama, me envía mensajes, se interesa por saber cómo estoy, y eso es bueno.* (UCI-10)

*En este centro nunca me han tratado con indiferencia, pero en el primer* [centro] *al que acudí para recibir atención, sí. Fui con una asistente social para realizar mi inscripción, y ella no me escuchó, no se preocupó por saber dónde era más cercano para que me atendieran. Me envió a este centro, con un papel de derivación incorrecto, y además me dio un pañuelo de papel y me dijo que me pusiera otra mascarilla, cuando ya llevaba dos.* (UCI-20)

Por esta razón, la acogida de los usuarios y la comprensión de su realidad por parte de los profesionales contribuyeron a caracterizar la atención humanizada. Al interpretar la atención humanizada desde la perspectiva de la atención prestada por el profesional, lo describieron como alguien respetuoso, bondadoso y que no confunde su vida personal con la profesional:

*Para que este tipo de atención se produzca, es necesario contar con un buen profesional, porque hay algunos que mezclan sus problemas personales con el trabajo y no logran brindar una atención humanizada.* (UCI-13)

[...] *es necesario que haya profesionales que entiendan y comprendan la situación de las personas que acuden en busca de salud, porque no todos atienden y acogen bien.* (UCI-18)

### Clase 3 – Obstáculos para la Práctica de la Humanización

En esta clase, los radicales que indicaban las palabras más significativas cobraron sentido en las UCE que revelaron obstáculos para la práctica de la humanización, como las limitaciones en la estructura física de las unidades de salud y la falta de recursos materiales, que comprometían el trabajo de los profesionales y, por consiguiente, la atención a los usuarios:

*El espacio es pequeño para atender a la gran cantidad de pacientes. No hay sillas para todos y hace mucho calor.* (UCI-11)

*Es necesario mejorar la estructura* física *del edificio y los recursos materiales para que los profesionales puedan trabajar mejor y, así, los pacientes se vean beneficiados.* (UCI-22)

Los participantes comprendían que la mejora de los servicios dependía de las inversiones de los gestores públicos, incluidas las mejoras en el espacio físico y la compatibilidad de los recursos humanos con respecto al número de usuarios, acercándose al conocimiento reificado para elaborar sus RS:

*Lo que aún debe mejorar está relacionado con la responsabilidad del municipio. Por ejemplo, contratar más médicos para atender y mejorar el espacio, que aún es pequeño, para una población que aumenta cada día.* (UCI-I7)

*Creo que el gobierno podría invertir* más para *mejorar la estructura* física y *la seguridad del centro, ampliar el espacio y contratar más profesionales para atender.* (UCI-23)

Teniendo en cuenta la rutina de las acciones para controlar la TB en estas unidades, afirmaron que la adherencia al tratamiento dependía de la forma en que eran atendidos por los profesionales, de modo que, si se sentían menospreciados o eran tratados con prejuicios, abandonarían el tratamiento y lo buscarían en otro lugar, lo que pone de manifiesto el consenso general para juzgar la viabilidad de dicha adherencia:

*Si aquí percibiera algún comportamiento prejuicioso por parte de los profesionales, sin duda no volvería y abandonaría el tratamiento o lo continuaría en otro lugar.* (UCI-22)

*Si recibiera un mal trato, sin duda habría abandonado el tratamiento aquí, buscaría otro lugar o tomaría medicamentos caseros.* (UCI-27)

## DISCUSIÓN

Los testimonios revelaron que los participantes, en su intento de explicar el fenómeno, representaron la humanización a partir de las relaciones interpersonales, basadas en la idea de una buena atención, y también a partir de lo que sería su opuesto, la «deshumanización», que se basaba en las experiencias cotidianas, incluidos los problemas estructurales de las unidades de salud en las que eran atendidos, donde debería existir una mayor preocupación por parte de la administración pública para hacer efectiva la PNH. En este contexto, se identificó una estrecha relación entre el conocimiento reificado y el universo consensual.

Evidenciado por el perfil de los participantes, la pertenencia grupal constituye un elemento importante de los estudios basados en la TRS, ya que permite comprender el contexto a partir del cual expresan sus representaciones, lo que Jodelet denomina «mundo de vida»^(17)^. Se verificó, por ejemplo, que el grupo de edad predominante se acercaba a los resultados del Boletín Epidemiológico de TB, publicado en 2024, que señalaba los grupos de edad de 20 a 34 y de 35 a 49 años como mayoritarios en el sexo masculino, y de 20 a 34 años en el sexo femenino^(3)^. La prevalencia entre los hombres sigue la tendencia global de la tuberculosis^(1,3)^, corroborada también por otros estudios^(13,14)^.

Los datos sobre el desempleo indican un panorama preocupante, ya que el tratamiento es largo, costoso y requiere una alimentación adecuada. Para quienes no tienen ingresos, puede ser aún más difícil satisfacer las necesidades fisiológicas que surgen de la enfermedad y del proceso terapéutico, incluidas las necesidades nutricionales, lo que puede dar lugar al abandono del tratamiento o a una respuesta insuficiente del organismo al mismo, debido a la desnutrición. En vista de ello, en el territorio brasileño, el Ministerio de Salud ha promovido iniciativas de protección social para las personas y familias afectadas por la TB, considerando la estrecha relación entre pobreza y enfermedad. Así, lleva a cabo acciones intersectoriales que involucran la salud, la educación y la asistencia social, con el objetivo de garantizar el bienestar de los afectados a partir de la integralidad de la asistencia^(6)^.

Las acciones inherentes a la salud y la educación se desarrollan a través de prácticas de atención realizadas por equipos multidisciplinarios. Estas prácticas deben articular la atención a las personas con TB con la atención a aquellos con quienes comparten la enfermedad (familiares, cuidadores y acompañantes), centrándose en aspectos como el tratamiento, la prevención de complicaciones, la rehabilitación y la reinserción social de los afectados, pero también en la búsqueda activa, la evaluación y el seguimiento de las condiciones clínicas, la prevención de la enfermedad y, si es necesario, el diagnóstico, el tratamiento y la rehabilitación entre los contactos^(2)^.

Dependiendo de las necesidades biopsicosociales de los afectados y de sus contactos, esto requiere la participación conjunta de dichos sectores, ya que se sabe que el nivel y las condiciones educativas influyen en la historia natural de enfermedades socialmente determinadas, como la TB, favoreciendo o reduciendo la disposición y las posibilidades individuales y colectivas para comprender e incorporar prácticas de autocuidado y prevención, convirtiéndolas en elementos que ratifican la importancia de la educación en salud. Además, es a través de la educación que los recursos humanos se califican para actuar en el control de la TB, razón por la cual se deben proponer y llevar a cabo acciones sistemáticas para estimular la educación continua y permanente, con el fin de subsanar las limitaciones que se derivan del trabajo en salud o atenuar sus efectos^(2)^.

Tejer esta explicación a la luz de la TRS implica recordar que las RS se inscriben en un contexto preexistente repleto de tradiciones, valores y creencias que están anclados al objeto que se representa, el cual se incorpora a las explicaciones cotidianas del individuo y de su grupo. Esta composición o intercambio de conocimientos/experiencias es imprescindible para construir pensamientos basados en el universo consensuado y compartirlos con el grupo, lo que brinda oportunidades de pertenencia social^(16)^.

El acceso a los programas sociales de apoyo y transferencia de ingresos, como la Pensión para Personas de Bajos Ingresos, el Beneficio de Prestación Continua, el Programa Bolsa Familia, el Programa Minha Casa/Minha Vida y la Tarifa Social de Energía Eléctrica, vigentes en Brasil, puede mitigar las repercusiones socioeconómicas de la TB en el presupuesto familiar y fortalecer la calidad de vida^(21,22)^. Aunque no son específicos para personas con la enfermedad, se entiende que el conocimiento sobre estos programas y la posibilidad de implementarlos son fundamentales para mejorar la asistencia, sobre todo a las personas de bajos ingresos^(23)^. Con el objetivo de afrontar colectivamente la TB, esto refuerza la articulación que debe existir entre el SUS y el Sistema Único de Asistencia Social (SUAS), tal y como reiteraron el Ministerio de Salud y el Ministerio de Ciudadanía al publicar la Instrucción Operativa n.º 1, de 26 de septiembre de 2019^(24)^.

En lo que respecta a las RS, basadas en un conjunto de categorías del sentido común y en la comprensión del proceso terapéutico como bueno o malo, los participantes construyeron sus conocimientos sobre la humanización a partir de oposiciones, y dicha comprensión se organizó a través de la realidad vivida en la atención recibida. Esto se debe a que los paradigmas almacenados en la memoria social establecen una relación positiva o negativa con el contenido que le es inherente, al que se accede a partir de la actividad cognitiva^(16)^.

La palabra «humanización» presenta una difícil comprensión conceptual, pero, cuando se asocia a las experiencias en los servicios de salud (dimensión de la información), los participantes demostraron comprender que tratar bien al otro y optar por una relación fructífera (dimensión de la actitud) genera comportamientos positivos en el contexto social (dimensión de la imagen). Así, se identifican las tres dimensiones de las RS^(16)^, estructurando el fenómeno de la humanización asociado a la atención asistencial.

En este contexto, los participantes materializaron la humanización a partir de la buena atención que deben brindar los profesionales, caracterizada por elementos como la escucha cualificada, la acogida, la empatía y el respeto, ampliamente deseados en el día a día de los servicios en todos los niveles de atención (primaria, secundaria/especializada y terciaria/hospitalaria). Otras investigaciones mostraron resultados similares, cuando los usuarios vincularon la humanización a las buenas prácticas del equipo de salud, anhelando ser tratados con cariño, educación y buen humor, mencionados como acciones que identificaban, en su concepción, una buena atención^(25,26)^.

La cognición y el afecto se entrelazan en la construcción del conocimiento y en los procesos de aprendizaje, ya que el desarrollo humano está mediado por la cultura, así como por las emociones y las interacciones sociales^(27)^. Por lo tanto, se admite que las conexiones entre los aspectos cognitivos, afectivos y sociales influyen en la construcción de conocimientos que producen significados sobre un objeto determinado^(16,17)^, motivo por el cual, en este estudio, las RS transitaron dialécticamente entre la deshumanización y la humanización de la atención a las personas con TB, ya que se citaron diversos elementos de la PNH, ya sea por su presencia o por su ausencia.

Por esta razón, dialogar sin arrogancia es una condición necesaria para que se establezcan relaciones horizontales, lo que facilita la comprensión de la información compartida entre las personas. Esto está contemplado en la PNH y, por lo tanto, debe generar el conjunto de acciones conocido como acogida, esencial para las relaciones de cuidado, ya que abarca, entre otros aspectos, el intercambio de información y el vínculo humano para orientar decisiones pertinentes y oportunas^(8)^. Del mismo modo, otros estudios señalan las relaciones acogedoras como una expresión humanizada que facilita la creación de vínculos y el sentimiento de confianza, ayudando a resolver los problemas cotidianos^(8,28)^.

Bajo este entendimiento, los participantes objetivaron al profesional de la salud como alguien respetuoso, bondadoso y que no confundía su vida personal con la vida que llevaba en su oficio, desde la perspectiva de ser alguien que acogía y era capaz de satisfacer los anhelos de los usuarios. La modificación de lo abstracto, de la idea-concepto, al modelo figurativo se configura como parte de la objetivación, y este modelo tiene como función presentar un punto común entre los diferentes conocimientos, traducir la representación real del objeto y asociar los elementos en una dinámica que le es propia^(16)^.

El largo período de tratamiento de la TB y el seguimiento en la APS permiten crear vínculos horizontalizados, pero es fundamental que los profesionales y los usuarios se comprometan a fortalecer sus relaciones interpersonales, que deben basarse en cinco pilares: cordialidad, ética, empatía, asertividad y autoconocimiento. El pilar de la cordialidad se refiere al hecho de que alguien sea amable y solícito, mientras que el de la ética se refiere al sentido de la justicia, el principio y la disciplina, siendo ambos necesarios para mejorar la atención, haciéndola resolutiva ante los problemas de salud, en este caso, la TB^(29)^.

El pilar de la empatía es inherente al acto de ponerse en el lugar del otro, estando dispuesto a escucharlo, contexto en el que los resultados de esta investigación mostraron que la escucha cualificada es un componente esencial de las prácticas asistenciales^(29)^. La acción de escuchar al otro puede ocurrir en varios momentos, como en la recepción, que propicia el diálogo oportuno entre el profesional y el usuario, adecuado a sus necesidades, permitiendo la evaluación de vulnerabilidades, gravedad y riesgos, de manera que favorezca el compromiso y el vínculo entre estos actores. De este modo, es posible ampliar la eficacia de las prácticas sanitarias, contribuyendo a la clínica ampliada, al favorecer decisiones compartidas y comprometidas con la autonomía de los usuarios del SUS^(5)^.

Desde esta perspectiva, llamó la atención la representación de la asistencia humanizada a través de dos acciones cognitivas humanas: «comprender» y «entender», mencionadas por UCI-18 en la clase 2. Comprender es un proceso continuo; por lo tanto, al tratar este concepto, se admite que no hay algo definido e inmutable, ya que lo que existe es una comprensibilidad en constante transformación. Entender, por su parte, consiste en escuchar, percibir y asimilar el propósito de algo^(30)^.

De esta manera, comprender implica una acción muy envolvente, impregnada de compromiso con el conocimiento de los aspectos más profundos del sujeto, mientras que entender configura un proceso más superficial y derivado de la palabra «comprender». Interpretar la asistencia humanizada a través de ambas palabras evidencia que este tipo de asistencia se reviste de características propias de la interacción, y aunque la estructura institucional sea sofisticada y el ambiente sea acogedor, nada supera la presencia del profesional, ya que solo un ser humano es capaz de comprender a otro^(30)^. Por lo tanto, cuando los usuarios se esfuerzan cognitivamente por representar la asistencia humanizada a la luz de sus experiencias, la comprensión se eleva como un elemento destacado que traduce la relación humana, tan necesaria para el cuidado^(31)^.

Para comprender al otro, es necesario establecer contacto para ayudarlo a superar las adversidades que pueden interferir en el cuidado de sí mismo, en el tratamiento y en la curación. Cambiar las perspectivas sobre los hábitos de vida que enferman o que ralentizan la curación requiere, a menudo, la necesaria (re)clasificación de las acciones cotidianas, incluso en las relaciones y las inserciones sociales, lo que exige que el profesional establezca vínculos de confianza con el usuario para que, en la construcción de un diálogo horizontal, la atención sea efectiva^(5,30)^.

El pilar de la asertividad fortalece las relaciones interpersonales cuando se trata de las interacciones entre el profesional y el usuario, basadas en una comunicación clara, directa y respetuosa. A su vez, el pilar del autoconocimiento permite al profesional comprender su personalidad para evitar o mitigar conflictos^(29)^, como se indica en los resultados de este estudio, cuando los participantes reconocieron que es importante separar los aspectos de la vida personal de los de la vida profesional.

En lo que respecta a la humanización, cabe destacar que, sin iniciativas adecuadas de educación permanente, no existe una calidad satisfactoria de las prácticas asistenciales^(5)^. Por lo tanto, en referencia a la necesidad de que los problemas personales no interfieran en la buena atención, los participantes señalaron que los profesionales no solo deben tener capacidad técnico-científica, sino también competencias y habilidades relacionales para, incluso, combatir acciones deshumanizadas, que repercuten en la capacidad de comprender al usuario.

Otro aspecto importante se refiere a los elementos del entorno, ya que influyen fuertemente en la asistencia y, por consiguiente, en el tratamiento y la curación. Al analizar la PNH, se observa que el entorno es un instrumento humanizador, ya que debe ayudar a promover el confort, la seguridad y el bienestar, además de ser un elemento que integra al equipo multidisciplinario, a los usuarios y a sus familiares^(25)^.

Teniendo en cuenta las especificidades de la TB, el Ministerio de Salud recomienda que, en la APS, se realice una planificación arquitectónica adecuada de las unidades de salud para evitar la propagación de *Mycobacterium tuberculosis* y otros agentes patógenos transmitidos por vía aérea, valorando aspectos como la iluminación y la ventilación adecuadas, ya que, en el caso de la TB, los bacilos expulsados por las personas con la enfermedad pueden dispersarse rápidamente^(2)^. Por lo tanto, es fundamental que haya espacios cuyas características garanticen la comodidad de estas personas y la seguridad sanitaria de los demás grupos que los frecuentan, lo que permite romper la cadena de transmisión.

El municipio de Belém se caracteriza por un clima cálido y húmedo, con períodos de poca o mucha lluvia^(32)^. Por ello, los espacios reducidos en las unidades de APS, sobre todo en las actividades cotidianas de control de la TB, pueden interferir significativamente en la salud humana, ya que los ambientes cerrados favorecen la dispersión de bacilos^(2)^ y la circulación de partículas y pequeños organismos, como el polvo y los ácaros^(33)^.

Según la PNH, el concepto de ambiente incorpora necesariamente espacios acogedores, cómodos y que garanticen la privacidad de los grupos humanos, pero que también favorezcan, siempre que sea necesario, cambios en el proceso de trabajo, promoviendo encuentros basados en el diálogo^(5)^. Así, estos espacios deben ofrecer a los profesionales y usuarios un ambiente adecuado e interactivo.

En contraste con esta información, los testimonios revelaron la imagen de un espacio pequeño y con pocas sillas, en cantidad insuficiente para atender la demanda de quienes buscaban atención. Esta realidad dificultaba el proceso de trabajo multiprofesional y las relaciones afectivas, reduciendo el bienestar de los usuarios en el contexto asistencial.

Se hace esta consideración porque, además de las representaciones que circulan sobre la TB, existen los retos a los que se enfrentan los sujetos. Estos desafíos se presentan de manera acumulativa y dan lugar a aspectos negativos y/o positivos frente a las experiencias en torno a la enfermedad, lo que permite construir nuevas miradas y significados que serán beneficiosos o perjudiciales, fortalecedores o limitantes, necesarios o innecesarios para la vida cotidiana y para enfrentar la TB, dependiendo de la naturaleza de sus contenidos^(34)^.

Así, los participantes señalaron a la gestión pública como responsable de proporcionar mejoras en los servicios, mediante inversiones destinadas efectivamente a la APS. En este sentido, reconocieron que los equipos de las unidades ponían en marcha acciones de salud, pero que correspondía a los gestores proporcionar los recursos necesarios. Por esta razón, se entiende que la falta de recursos básicos para calificar los servicios se caracterizó como una condición inhumana, ya que repercutía en la salud tanto del profesional como del usuario.

Esto significa que la deshumanización puede evidenciarse no solo a través de actitudes y comportamientos, sino también por la falta de estructura y recursos, que obstaculizan la adherencia terapéutica entre las personas con TB^(28)^. Esta comprensión ayuda a explicar la decisión de los participantes de buscar otros servicios u optar por otras formas de tratamiento, en caso de que la atención recibida en las unidades fuera irrespetuosa o presentara indicios de prejuicio.

Desde esta perspectiva, el diálogo entre el conocimiento reificado y el universo consensuado permite la dinámica de las RS, contexto en el que los cambios de concepción y la unión de los conocimientos que provienen de ambas lógicas de pensamiento son fundamentales para (re)construir estas RS. También permite comprender cómo el conocimiento científico es apropiado por las personas en el discurso social y se convierte en contenido común del saber popular, proporcionando elementos para caracterizar las formas en que los grupos sociales piensan y actúan ante un objeto o contexto con importancia subjetiva^(35)^.

Este resultado destaca que las RS sobre la atención humanizada, en el contexto de la TB, se basan en la idea de una buena atención como referencia para mantener al usuario en seguimiento. Este es un elemento importante, ya que demuestra que las inversiones adecuadas en la formación técnico-científica y relacional de los profesionales son importantes para efectuar el tratamiento^(2)^.

Para la PNH, humanizar la atención significa valorar a los diferentes sujetos implicados en el proceso de producción de la salud (usuarios, trabajadores y gestores), incluyendo valores como la autonomía y el protagonismo, la corresponsabilidad, el establecimiento de vínculos solidarios y la participación colectiva en el proceso de gestión^(31)^. Un sistema de salud que funcione adecuadamente, con gestión participativa, financiamiento, mano de obra y recursos tecnológicos, estimula el progreso de la APS^(36)^.

Por lo tanto, es necesario calificar la PNH y ampliar las formas de actuar de los profesionales que trabajan en ella, construyendo alternativas que valoren las diferencias, las singularidades y las prácticas humanizadas, que deben sustentarse en actitudes basadas en el respeto mutuo, pero también en acciones centradas en el compromiso colectivo. Cabe destacar que, en el ámbito de la salud, los cambios estructurales y socioculturales, orientados a la calidad y la humanización de la asistencia, pueden convertirse en elementos importantes en el proceso de ampliación del campo simbólico de las RS sobre la humanización^(37)^.

Las limitaciones de este estudio corresponden a su realización en un municipio específico de un estado brasileño, por lo que el alcance de sus resultados se restringe a escenarios con características similares o aproximadas, lo que hace necesario desarrollar otros estudios de SR sobre el tema, a fin de contemplar los tres niveles de atención. Por lo tanto, es necesario comprenderlo en otros espacios sociales, ayudando a construir y difundir reflexiones basadas en la valoración igualitaria del conocimiento reificado y del universo consensual, lógicas de pensamiento que permiten identificar las RS.

Ante sus resultados, este estudio contribuye a las áreas de enfermería y salud colectiva, en la medida en que puede subsidiar el diálogo fructífero entre estudiantes, profesionales, gestores y autoridades públicas para calificar los servicios de salud y proponer nuevas estrategias que fortalezcan la humanización en el día a día de la asistencia a las personas con TB.

Esta consideración se hace teniendo en cuenta que, en el contexto práctico, estos actores sociales pueden ser estimulados a pensar críticamente sobre los factores que obstaculizan la práctica de la humanización en los servicios de APS. Un ejercicio reflexivo coherente puede orientarlos en la propuesta de medidas que repercutan en los diferentes elementos que implican la humanización, entre los que destacan las características estructurales de las unidades de salud, los procesos de trabajo multiprofesional y las relaciones humanas que se tejen en ellas con los usuarios, como se señala en los testimonios. Para que sean eficaces y fortalezcan la humanización, estas medidas deben tener en cuenta los retos administrativos, materiales, operativos, políticos y socioculturales inherentes a estos servicios, con el fin de transformar en cierta medida sus realidades.

## CONCLUSIÓN

Se evidenció que la humanización de la asistencia se basó en las relaciones interpersonales establecidas entre los profesionales de la salud y los usuarios, de manera que se sustentaran en el sentimiento de confianza y el buen trato, característicos de una atención calificada. Sin embargo, en la realidad de los participantes, se puede inferir que no se concretó, debido a las limitaciones en la estructura física de las unidades de salud donde eran atendidos y a la falta de recursos materiales, reconocidos como problemas inherentes a la gestión pública, que debían superarse para fortalecer las acciones de control de la TB en la APS.

En este sentido, las relaciones interpersonales constituyeron el pilar de la humanización, ya que a través de ellas se facilitaba el entendimiento mutuo sobre la enfermedad, el proceso terapéutico y las adversidades que podían surgir en este contexto, lo que favorecía la adherencia al tratamiento. Así, conocer los elementos que constituían o influían en las RS de las personas con TB sobre el tema demostró la importancia de las relaciones humanas en las actividades asistenciales, sobre todo ante los retos del trabajo multiprofesional en la APS, incentivando la construcción y el fortalecimiento del vínculo entre equipos y usuarios.

Ante las RS aquí reveladas, se entiende que nuevas investigaciones pueden ayudar a comprender otros aspectos psicosociales en torno a la humanización, lo que se traduce en contribuciones a la asistencia, la gestión de los servicios, la enseñanza y la investigación en el campo de la salud. Estas esferas de poder y actuación sociopolítica son inherentes a diversos profesionales, entre ellos los enfermeros, cuyas responsabilidades éticas, sociales y técnicas guían la identificación de las necesidades de los grupos humanos para aplicar intervenciones que transformen, en cierta medida, la realidad que viven, permitiendo incorporar prácticas de atención individualizada.

## Data Availability

Los datos que respaldan este estudio están disponibles a solicitud del autor correspondiente.
